# Overcoming the blood‒brain barrier: nanomedicine strategies for targeted delivery and multimodal therapy in Alzheimer's disease

**DOI:** 10.1080/10717544.2026.2645830

**Published:** 2026-03-20

**Authors:** Jiahui Li, Liting Guo, Weiyi Cai, Juanjuan Mei, Jie Liu, Yanan Liu

**Affiliations:** a Department of Central Laboratory, Shenzhen Longhua District Maternity and Child Healthcare Hospital, Shenzhen, China; b Department of Pathology, Shenzhen Longhua District Maternity and Child Healthcare Hospital, Shenzhen, China; c Department of Chemistry, College of Chemistry and Materials Science, Jinan University, Guangzhou, China

**Keywords:** Alzheimer's disease, nanomedicine, nanoparticles, blood–brain barrier, drug delivery, amyloid-β, tau protein, reactive oxygen species

## Abstract

Alzheimer's disease (AD) remains a significant therapeutic challenge, primarily because the formidable blood‒brain barrier (BBB), which drastically limits the brain bioavailability of most drugs. Nanoparticle-based drug delivery systems offer a promising strategy to overcome this central obstacle. This review systematically examines the design, mechanisms, and applications of nanomedicine in AD therapy. We analyze key strategies for enhancing BBB penetration through surface engineering and the utilization of various nanocarriers, including liposomes, exosomes, dendrimers, and carbon dots. Furthermore, we discuss how stimuli-responsive release mechanisms (e.g. responsive to pH, enzymes, reactive oxygen species, light, or ultrasound) enable targeted and precise drug delivery. A critical focus is placed on how these multifunctional nanoplatforms can address multiple AD pathogenic pathways simultaneously, such as amyloid-β and tau aggregation, cholinergic dysfunction, oxidative stress, neuroinflammation, and gut‒brain axis dysregulation. Although preclinical evidence is compelling, the clinical translation of these nanotherapies is hindered by challenges related to long-term biocompatibility, scalable manufacturing, patient heterogeneity, and regulatory frameworks. This review highlights the translational potential of nanomedicine in AD treatment while outlining the key hurdles that must be addressed for its successful implementation.

## 1 Introduction

Global aging has intensified rapidly, and medical advances have made China one of the world's fastest-aging nations, with an aging rate exceeding that of North America and Europe (Liu et al. 2022). By 2023, 280 million Chinese (19.84% of the population) were aged ≥60 years, and 210 million (14.86%) were aged ≥65 years, marking entry into a deeply aged society and imposing heavy societal and familial burdens. Alzheimer's disease (AD), a leading cause of mortality in elderly individuals (Jia et al. 2020), is the most prevalent form of dementia and a major neurodegenerative disorder characterized by language impairment, memory loss, mood disorders (e.g. depression, anxiety), and life-threatening complications in advanced stages. AD and related dementia constitute the fifth leading cause of death in China, which bears a disproportionate global burden with higher age-standardized prevalence, incidence, and mortality than the worldwide average (Ren et al. 2022; Zhi et al. 2025). Timely diagnosis, treatment, and early screening of AD are therefore urgent public health priorities.

AD is a complex neuropathological disorder defined by two core hallmarks: extracellular amyloid-β (Aβ) plaque accumulation and intraneuronal aggregation of hyperphosphorylated tau (*τ*) protein into neurofibrillary tangles (NFTs) (Hyman et al. 2012). The major pathogenic hypotheses include the amyloid cascade and tau protein hypotheses. Clinical diagnosis relies on detecting biomarkers in cerebrospinal fluid (CSF), including total tau, hyperphosphorylated tau, Aβ_42_ levels, and the Aβ_42_/Aβ_40_ ratio, as well as imaging of tau/Aβ deposits via positron emission tomography (Hyman et al. 2012).

AD is classified into familial early-onset AD (EOAD, <5% of cases, age at onset <65 years) and sporadic late-onset AD (LOAD, the majority of cases, age at onset ≥65 years) (Long and Holtzman 2019). EOAD is strongly associated with genetic mutations: point mutations in the amyloid precursor protein (*APP*) gene (chromosome 21, Val → Ile) (Goate et al. 1991), missense mutations in presenilin-1 (*PSEN1*) (e.g. S182) (Sherrington et al. 1995), and point mutations in presenilin-2 (*PSEN2*) (e.g. N141I) (Levy-Lahad et al. 1995). For LOAD, the apolipoprotein E ε4 allele (*APOEε4*) on chromosome 19 is the major genetic risk factor; its homozygous form is present in nearly all AD patients over 80 years of age, impairing cerebral Aβ clearance and promoting deposition (Corder et al. 1993; Blanchard et al. 2022). Other risk genes include β-site amyloid precursor protein cleaving enzyme 1 (*BACE1*, chromosome 11, which mediates amyloid accumulation) and triggering receptor expressed on myeloid cells 2 (*TREM2*, which regulates microglial function in Aβ or tau pathology) (Jonsson et al. 2013; Qin et al. 2016, 2021, which may modulate AD susceptibility through inflammatory pathways. Aging is the primary risk factor for LOAD, which arises from multifactorial mechanisms and shows a trend toward earlier onset (Ren et al. 2022). Additional pathogenic mechanisms include cholinergic deficits, mitochondrial dysfunction accompanied by oxidative stress, neuroinflammation, and gut–brain axis dysbiosis (Abdallah 2024). Overall, AD is a neurodegenerative disease that affects the central nervous system (CNS) and is driven by complex gene–environment interactions.

Current AD therapies include oral small-molecule drugs and immunotherapy. The FDA has approved acetylcholinesterase (AChE) inhibitors, including donepezil (1996), galantamine (2001), rivastigmine capsule (2000), and rivastigmine transdermal patch (2007), for the treatment of AD, as well as the N-methyl-D-aspartate (NMDA) receptor antagonist memantine for moderate-to-severe AD (Donepezil et al. 2018). These agents only provide symptomatic relief with limited efficacy, highlighting the urgent need for disease-modifying therapies. The FDA approved aducanumab (a humanized anti-Aβ monoclonal antibody representing the first disease-modifying immunotherapy for AD and mild cognitive impairment [MCI]) in 2021 and lecanemab via accelerated approval in 2023 (Parums 2021; Sha et al. 2026). However, current immunotherapies target single pathological mechanisms and exhibit limited efficacy against the multifactorial pathology of AD.

Given these limitations, bioactive compounds (e.g. polyphenols, flavonoids, and terpenoids) have attracted extensive interest for AD prevention and treatment. For example, the wheat- and barley-derived polyphenol AR-C17 exerts cognitive-improving effects in *APP/PSEN1* mice (Liu et al. 2020); the flavonoid quercetin (QUE) shows neuroprotective potential (Gonzales et al. 2023); and the representative terpenoid ginsenoside Rg1 ameliorates AD-related pathologies (Wu et al. 2022). Despite promising therapeutic effects, their clinical application is limited by large molecular size, hydrophobicity (impairing blood‒brain barrier [BBB] penetration), complex metabolism, poor bioavailability, susceptibility to degradation, and short half-lives (Zhu et al. 2022; Xia et al. 2024). Enhancing their bioavailability and stability thus represents a key research priority.

Recent advances in nanomedicine have enabled the engineering of nanoparticles (NPs)—biomolecular-scale, cell-interactive structures with excellent biocompatibility, high drug-loading efficiency, tunable pharmacokinetics (prolonged half-life), and controlled or sustained release. These properties support targeted activation of endogenous repair and immune responses, advancing disease diagnosis, drug and gene delivery, and therapeutic intervention (Boulaiz et al. 2011). Representative applications include azide-labeled T-cell-membrane-biomimetic NPs for tumor targeting and diagnosis (Han et al. 2019); CTCE9908 peptide-loaded silica-crosslinked micelles modified with near-infrared (NIR) probes for theranostics of CXCR4-overexpressing liver fibrosis (Wang et al. 2024); green-synthesized silver nanoparticles with anticancer, hypoglycemic, and antibacterial activities via mechanisms including mitochondrial repair, DNA damage, apoptosis, and enzyme inhibition (Javed et al. 2021); and polymeric NPs for drug delivery across the BBB in Parkinson's disease (PD) (He et al. 2025).

NPs show great promise for AD diagnosis: magnetic nanoparticles (MNPs) serve as high-efficiency magnetic resonance imaging (MRI) contrast agents, which can be engineered via surface modification or prepared as nano metal-organic frameworks (Zhao et al. 2020; Chaparro et al. 2023); fluorescent poly(lactic-co-glycolic acid) (PLGA) NPs can identify Aβ plaques and Congo red-stained neuritic plaques in the cortex of AD mouse models (Govindarajan and Kar 2023); phenylenediamine-derived red-emissive carbon dots (CDs) bind to Aβ aggregates through π-conjugation, hydrogen bonding, and hydrophobic interactions (Wei et al. 2024). Critically, NPs can cross the BBB for targeted therapeutic delivery, demonstrating significant potential for treating neurodegenerative diseases—supported by approximately 660 relevant publications on PubMed (2005–present) related to *nanoparticles* and *Alzheimer's disease*.

Traditional AD therapies suffer from low bioavailability and poor targeting due to the BBB, and nanoparticle (NP)-based drug delivery systems offer a revolutionary brain-targeted strategy through designable physicochemical properties. This review systematically outlines the rationale and design strategies of such nanosystems. First, surface engineering to enhance BBB penetration (charge modification, ligand conjugation), along with the design, advantages, and limitations of major nanocarriers (liposomes, exosomes, dendrimers, cell–membrane mimetics, CDs); second, intelligent stimulus–responsive drug release systems that exploit the AD microenvironment (specific pH, enzymes, reactive oxygen species [ROS]) or external stimuli (light, ultrasound, electric fields) for precise controlled release; third, synergistic nanoplatforms targeting multifactorial AD pathology (inhibition of Aβ/tau aggregation, modulation of the cholinergic system, alleviation of oxidative stress and neuroinflammation, and regulation of the gut microbiota); and finally, clinical translation bottlenecks (patient heterogeneity, long-term safety, scalable manufacturing, regulatory frameworks) and future perspectives. This review aims to provide a foundation for the clinical translation of nanomedicine in early screening, intervention, and treatment of AD.

## 2 Delivery systems of nanomedicine for AD treatment

AD therapy remains a major challenge, as current clinical agents suffer from inadequate brain bioavailability owing to poor aqueous solubility and limited tissue permeability. Optimized drug delivery routes are thus essential for brain-targeted AD treatment, and NP-based delivery systems represent a promising strategy to deliver therapeutics to pathological sites, thereby inhibiting Aβ aggregation and tau neurofibrillary tangle formation. Nanomedicine fabricates NP-based pharmaceuticals via the assembly of active pharmaceutical ingredients (APIs) with nanocarriers for disease diagnosis and treatment (Shan et al. 2022), yet its systemic delivery is hindered by poor biocompatibility, off-target toxicity, low tissue specificity and, most critically, an impermeable BBB.

**Figure 1.**
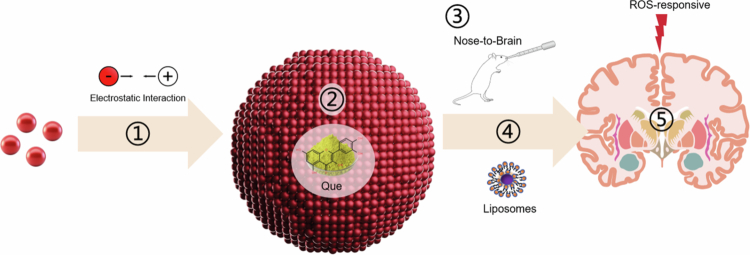
Schematic overview of nanoparticle-mediated drug delivery for AD therapy. The process involves: ① Nanoparticle self-assembly (e.g. via electrostatic interactions); ② Loading of active pharmaceutical ingredient (e.g. QUE); ③ Administration routes (e.g. nose-to-brain); ④ BBB penetration facilitated by engineered nanocarrier properties (e.g. liposomes); and ⑤ Stimuli-responsive drug release (e.g. ROS-responsive). Created with Bioicons.com and SciDraw.io.

The BBB is a key CNS defensive structure but a major barrier for treating neurodegenerative diseases, including AD and PD. Composed of endothelial cells, astrocytes, and microglia, it triggers immune clearance and impairs targeted drug penetration, reducing therapeutic efficacy (Shay et al. 2023). NPs (1–100 nm) loaded with APIs have been engineered to enhance BBB traversal via surface charge modification, ligand conjugation and hydrophilicity optimization (Song et al. 2023). This section elaborates on the core stages of NP-mediated brain delivery for AD (Figure 1): self-assembly, API loading, systemic administration, BBB penetration and stimuli-responsive release.

### 2.1 NPs self-assembly

**Figure 2.**
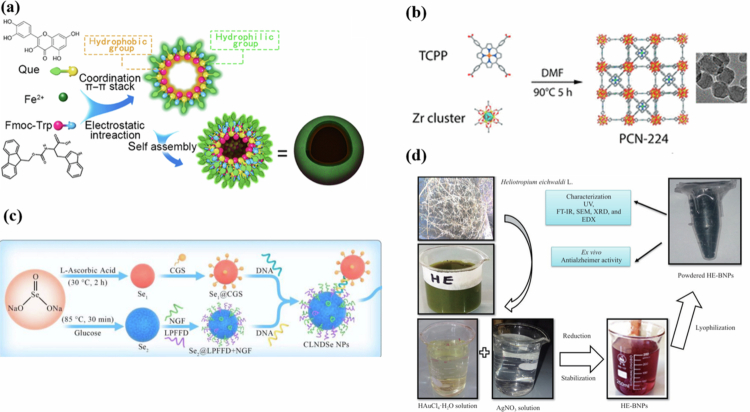
Schematic depiction of NP self-assembly processes. (a) Electrostatic interactions; (b) coordination bonding; (c) DNA-mediated double-strand complementary base pairing; (d) plant extract-based bio-green synthesis. Reprinted from Zhu et al. (2022) (Springer Nature, 2022), Wang et al. (2018) (American Chemical Society, 2018), Wang et al. (2023) (Elsevier, 2022), Sher et al. (2023b) (CC BY 4.0).

NP self-assembly is increasingly crucial for drug delivery. Encapsulating APIs within NPs leverages the unique structures and properties of the NPs. This approach reduces metabolic degradation of the APIs *in vivo* and enhances their targeting precision toward AD pathological features while allowing for controlled release kinetics. Self-assembly typically occurs via mechanisms such as electrostatic interactions, coordination bonding, DNA-mediated double-strand complementary base pairing, or plant extract-based bio-green synthesis (Figure 2).

Coordination-driven self-assembly yields Fmoc-Trp-Fe^2+^-quercetin (FTFQ) NPs (Zhu et al. 2022), with building block bioaffinity endowing enhanced biocompatibility, intermolecular interactions and degradation resistance; these NPs improve QUE bioavailability to inhibit Aβ aggregation and mitigate ROS/Aβ oligomer-induced neuronal cytotoxicity in AD cell and animal models. Similarly, porous coordination network-224 (PCN-224) NPs—hydrothermally synthesized using tetra-kis(4-carboxyphenyl)porphyrin (TCPP) as a zirconium ligand (Wang et al. 2018)—exhibit favorable photooxidation efficiency and structural stability, which blocks monomeric Aβ assembly into neurotoxic β-sheet-rich aggregates and reduces Aβ-induced neuronal damage linked to AD pathogenesis. Furthermore, Se-rutin, prepared via electrostatic in situ selenium reduction (Zhu et al. 2023), activates the nuclear factor erythroid 2-related factor 2 (*Nrf2*) antioxidant signaling pathway more potently than free rutin to alleviate AD-associated oxidative stress in preclinical models. Yet, the specific molecular mechanisms and optimal dosing of all three nanosystems remain unelucidated.

DNA-mediated self-assembly enables sequence-specific fabrication and precise API release control for AD therapy. A multifunctional double selenium NP system conjugates DNA, LPFFD peptide, and nerve growth factor (NGF) to selenium beads for A₂A adenosine receptor-mediated BBB delivery and has been reported to attenuate cognitive deficits in AD mouse models (Wang et al. 2023). Rabies virus glycoprotein 29 (RVG29)-modified DNA nanoframeworks (DFs) are engineered via rolling circle amplification for targeted miR-124 delivery across the BBB to ameliorate AD-related neuronal pathological damage (Ouyang et al. 2022). Yet, DNA nanostructures suffer from poor in vivo enzymatic stability and potential peripheral *A₂AAR* off-target effects that may trigger systemic side effects.

Plant extract-based bio-green synthesis of silver/gold bimetallic NPs (Ag/Au BNPs) using *Heliotropium eichwaldi L.* (HE) extract (Sher et al. 2023a, 2023b) features low toxicity and rapid reaction kinetics (24 h, 40 °C, pH 5, 1 mL HE extract), with Ag/Au BNPs exhibiting potent anti-AChE activity to target cholinergic dysfunction—a classic pathological feature of AD. However, batch-to-batch variability of plant extract components, uncharacterized central neurotoxicity and lack of standardized synthesis protocols severely limit their translational potential for AD treatment.

### 2.2 Loading of active pharmaceutical ingredients (APIs)

APIs are biologically active compounds that directly modulate bodily functions or structures for disease diagnosis, treatment, symptom relief or prevention, with examples including acetaminophen, acetylsalicylic acid, and amoxicillin (Park et al. 2021). Most APIs for targeted AD therapy are AChE inhibitors (e.g. donepezil, galantamine) or NMDA receptor antagonists (e.g. memantine). Despite their clinical efficacy, these agents induce adverse effects linked to oral administration (nausea, vomiting, diarrhea, fatigue, headache, and dizziness), while non-oral formulations (e.g. rivastigmine transdermal patches) may cause dermatological reactions or hepatorenal toxicity (Thangwaritorn et al. 2024). Thus, research has increasingly focused on novel APIs for AD, including natural antioxidants, neurotrophins, and neuropeptides (Table 1). These compounds exhibit low toxicity, improved bioavailability, and sustainable sourcing, rendering them promising candidates for early AD therapeutic intervention.

**Table 1.** Nanoparticles-mediated delivery of AD-targeting agents: natural antioxidants, neurotrophins, and neuropeptides.

Active ingredients	Structure type	Nanodrug delivery system	Mechanism of action	Development stage	Limitations		Reference
**Natural antioxidants**							
Quercetin (QUE)	Polyphenolic flavonoids	siB/QU@L-OL	Alleviated oxidative stress and neuroinflammation	Preclinical	No clinical validation; uninvestigated long-term efficacy; suboptimal targeting		Zhang et al. (2026)
		TQCN	Reduced iron overload and its induced free radical burst	Preclinical	Unclear long-term safety/metabolism; unoptimized dosage; no head-to-head clinical comparison		Liu et al. (2024)
Anthocyanins	Polyphenolic flavonoids	PLGA@PEG-An-NPs	Improved the neuroprotective effect and slowed the oxidative stress response	*In vitro*	Single model validation; suboptimal NP physicochemical properties; unclear mechanism		(Amin et al. 2017)
		PEG-AuNPs	Inhibited the p*-JN*K/*NF-κB*/p-GSK3β pathway	Preclinical	Low encapsulation efficiency; poor particle size uniformity		Kim et al. (2017)
Resveratrol (RSV)	Polyphenols non-flavonoids	RSV@SeNPs	Inhibited Cu^2+^ -induced Aβ aggregation and ROS generation	*In vitro*	Single *in vitro* model validation; no *in vivo* efficacy		Yang et al. (2018)
		RSV-loaded vesicles	Alleviated Aβ fibrillation, microglia dysfunction	Preclinical	Low encapsulation efficiency (49.67%); uninvestigated long-term safety		Jia et al. (2023)
		RVG/TPP-RSV NPs@RBCm	Reduced Aβ-associated mitochondrial oxidative stress	Preclinical	Incompletely elucidated targeting mechanism; uninvestigated long-term *in vivo* safety		Han et al. (2020)
		TGN-RSV@SeNPs	Reduced gut microbiome disorders	Preclinical	Unclear long-term metabolism; incomplete gut-brain axis mechanism		Li et al. (2021)
Curcumin (CUR)	Diketone compounds	LNCs	Played a neuroprotective role	*In vivo*	Unvalidated *in vitro* pharmacodynamics; unclear nanocarrier metabolism		Giacomeli et al. (2019)
		PDLC NPs	Enhanced anti-amyloid activity	Preclinical	Unvalidated *in vivo* efficacy; unassessed photothermal tissue damage		Yi et al. (2020)
Berberine (BBR)	Quinoline alkaloids	BR-CL and other nanoliposomes	Antagonized tau phosphorylation, inhibited AChE activity and neuronal apoptosis	Preclinical(phased research)	No *in vitro* cell experiments; uninvestigated dose-dependence		Wang et al. 2023
		MSNs-BBR-L	Inhibited AChE activity and amyloid fibrillation	Preclinical	Unclear long-term silica carrier metabolism; unvalidated tau intervention		Singh et al. (2021)
**Neurotrophins**							
Nerve growth factor (NGF)	Nuclear proteins	R@NGF-Se-Se-Ru	Repaired damaged nerves	Preclinical	Unclear ruthenium ion metabolism; unvalidated tau intervention		Yuan et al. (2021)
		NGF-WP5⊃Cys@Pd NCs	Removed ROS and protected NCs from oxidative damage	*In vitro*	Unclear *in vivo* metabolic mechanism; unvalidated brain targeting		Yuan et al. (2023)
		NGF-PCM@Ru NPs	Inhibited tau hyperphosphorylation, restored nerve damage, and maintained neuronal morphology	Preclinical	Unclear BBB penetration mechanism; unelucidated ruthenium metabolism		Zhou et al. (2020)
		NGF-PEG-PLGA-NPs	Promoted the differentiation of neural stem cell and protected basal forebrain cholinergic neurons	*In vitro*	Unclear *in vivo* metabolism; incomplete combination therapy mechanism		Chen et al. (2015)
**Neuropeptides**							
Melatonin	Indole heterocycles	DM-NC	Inhibited Aβ aggregation and misfolding	*In vitro*	Unvalidated long-term *in vivo* safety; suboptimal BBB penetration		Srivastava et al. (2020)
		MIT	Decreased tau phosphorylation and CKMT1 expression	Preclinical	Unvalidated nose-to-brain delivery; incomplete synergistic mechanism		Fihurka et al. (2023)

Abbreviations: siB/QU@L-OL, lipid nanoparticles (LNP) co-encapsulating *BACE1* small interfering RNA (siB) and quercetin (QU), surface-functionalized with Odorranalectin (OL)；NPs; TQCN, triphenylphosphine-modified QC nanoparticles; PLGA@PEG-An-NPs, encapsulated the anthocyanins in nanoparticles based on poly(lactic-co-glycolic acid) and polyethylene glycol (PEG)-2000; PEG-AuNPs, polyethylene glycol-gold nanoparticles; RSV@SeNPs, RSV-loaded selenium nanoparticles; RVG/TPP-RSV NPs@RBCm, rabies virus glycoprotein and triphenylphosphine cation molecules NPs encapsulating RSV attached to the RBC membrane surface; TGN-Res@SeNPs, transport peptide to generate Res-selenium-peptide nanocomposites; LNCs, curcumin-loaded lipid-lore nanocapsules; PDLC NPs, polymer-dispersed liquid crystal NPs; BR-CL, BR common nanoliposomes; MSNs-BBR-L, BBR loading, and lipid coating of mesoporous silica nanoparticles; R@NGF-Se-Se-Ru, NGF delivery using a ruthenium-based nano-platform; NGF-WP5⊃Cys@Pd NCs, loaded-NGF nanovalve system from palladium nanoclusters and carboxylated pillar[5]arene; NGF-PCM@Ru NPs, loaded NGF into ruthenium nanoparticles, and phase change material tetradecyl alcohol as a thermal response switch; DM-NC, Dopamine-Melatonin Nanocomposite; MIT, multi-target nano-agent comprising melatonin, insulin, and tetrahydrocannabinol; JNK, c-Jun N-terminal kinase; *NF-κB*, nuclear factor-kappa B; GSK3β, glycogen synthase kinase 3 beta; p-, phosphorylated; and CKMT1, creatine kinase mitochondrial 1.

#### 2.2.1 Natural antioxidants

AD pathology is characterized by Aβ_42_ aggregation, tau hyperphosphorylation, and neuroinflammation—all closely linked to oxidative stress–driven ROS overproduction. Extensive experimental evidence has demonstrated that natural antioxidants represent a major class of therapeutic agents for AD treatment. These include polyphenolic flavonoids (e.g. QUE, anthocyanins) and nonflavonoid compounds (e.g. resveratrol [RSV], curcumin [CUR], berberine [BBR]), which potently inhibit Aβ aggregation and alleviate oxidative stress.

QUE, a natural flavonoid, harbors intrinsic metal-chelating and antioxidant properties mediated by three metal-binding moieties in its catechol domain. Zhang et al. (2026) used QUE-loaded NPs to mitigate Aβ-induced oxidative stress and neuroinflammation via the activation of the phosphatidylinositol 3‑kinase/protein kinase B/nuclear factor erythroid 2‑related factor 2 (PI3K/Akt/Nrf2) signaling pathway. Liu et al. (2024) developed triphenylphosphine-modified QC nanoparticles (TQCNs), where QUE chelates iron via polyphenol coordination and self-assembles in situ into metal‒phenol nanocomposites. This system reduces brain iron overload and free radical bursts, reconstitutes the *Nrf2*-mediated antioxidant defense system, restores iron metabolic homeostasis, and enhances cytoprotective cascades (e.g. lipid peroxidation detoxification), thus providing a multifunctional strategy to target AD-associated ferroptosis.

Anthocyanins, a flavonoid subgroup, exhibit antioxidant, anti-inflammatory and neuroprotective activities, yet their phenolic hydroxyl group oxidation forms unstable quinones that impair bioactivity. Amin et al. (2017) encapsulated anthocyanins in PLGA and polyethylene glycol (PEG)-2000-based nanoparticles (PLGA@PEG-An-NPs), which enhanced the bioactivity and neuroprotection against Aβ-mediated oxidative stress in SH-SY5Y cells. Kim et al. (2017) further showed that anthocyanin-PEG-AuNP conjugates more potently suppress phosphorylated c-Jun N-terminal kinase (p-JNK)/*NF-κB*/phosphorylated glycogen synthase kinase 3β (p-GSK3β) pathway than free anthocyanins, while reducing Aβ-induced neuroinflammatory markers and neuronal apoptosis.

RSV is a promising antioxidant for AD but suffers from low *in vivo* bioavailability and aqueous solubility, leading to incomplete inhibition of Cu^2+^-induced Aβ_42_ aggregation at low concentrations. Yang et al. (2018) developed RSV-loaded selenium NPs (RSV@SeNPs), where SeNP modification synergistically inhibited Cu^2+^-induced Aβ_42_ aggregation and ROS generation and protected PC12 cells from Aβ-Cu^2+^-mediated cytotoxicity. Recent studies have confirmed that RSV-loaded NPs precisely intervene in Aβ fibrillation and microglial dysfunction and reduce Aβ-associated mitochondrial oxidative stress and gut microbiota dysbiosis, thereby alleviating AD pathology (Han et al. 2020; Li et al. 2021; Jia et al. 2023).

CUR, a natural turmeric-derived compound, possesses diverse bioactivities and neuroprotective effects, including Aβ aggregation inhibition. Giacomeli et al. (2019) encapsulated CUR in lipid-core nanocapsules, which enhanced neuroprotection against Aβ-induced behavioral and neurochemical deficits in aged female AD mice compared to free CUR. Yi et al. (2020) engineered phase-change NPs for NIR laser-triggered CUR release, which increased antiamyloid activity and reduced Aβ-induced cytotoxicity in PC12 cells.

BBR, an isoquinoline alkaloid from Berberis rhizomes, exerts neuroprotection by antagonizing tau hyperphosphorylation, inhibiting AChE activity and preventing neuronal apoptosis. To overcome its low bioavailability and systemic adverse effects, liposomal encapsulation is used for brain-targeted BBB delivery, which achieves low toxicity and high encapsulation efficiency to advance AD therapeutic strategies (Singh et al. 2021; Wang et al. 2023).

Despite promising preclinical efficacy, natural antioxidants face critical translational and safety limitations for AD clinical application, including a narrow therapeutic window and variable efficacy influenced by individual metabolism, genetic polymorphisms and comorbidities. Standardized clinical trials are thus urgently required to define dose-escalation protocols, long-term safety profiles and pharmacokinetic‒pharmacodynamic relationships. Individualized dosing strategies should also be considered, especially for AD patients with impaired hepatic or renal function (Ranjha et al. 2023).

#### 2.2.2 Neurotrophins

Neurotrophins are peptides or small proteins that support neuronal growth and survival, conferring neuroprotective effects in AD. Key members include NGF and brain-derived neurotrophic factor—the earliest discovered and most extensively studied neurotrophins—which are essential for maintaining nervous system homeostasis (Wei et al. 2024).

NGF, the first identified neurotrophin, preserves the integrity and function of cholinergic neurons. Impaired cholinergic signaling in the basal forebrain is closely linked to memory decline in AD, establishing endogenous NGF as a core therapeutic target (Mitra et al. 2019; Gavioli et al. 2024). NP systems have been engineered to enable targeted NGF delivery for AD. Yuan et al. (2021) developed a ruthenium-based platform (R@NGF-Se-Se-Ru) that promoted neuronal regeneration and nerve repair in AD models. The same group subsequently designed a supramolecular nanovalve system (NWP) comprising palladium nanoclusters (Pd NCs) and a carboxylated pillar[5]arene (WP5), which enabled precise NGF release. This platform scavenged ROS, protected neurons from oxidative damage, and polarized microglia toward the anti-inflammatory M2 phenotype, reducing neuroinflammation (Yuan et al. 2023). Zhou et al. (2020) engineered a nanocomposite of ruthenium NPs (Ru NPs) and phase-change material (PCM); stimulus-triggered NGF release inhibited tau hyperphosphorylation, reduced oxidative stress, restored neuronal morphology, and improved cognitive function in AD mice. Chen et al. (2015) further showed that NGF-loaded PEG-PLGA NPs enhanced neural stem cell differentiation *in vitro*, supporting combinatorial nanomedicine and cell-based therapy for AD.

Despite their potential, neurotrophins face critical limitations. Current investigations into their AD-related mechanisms remain incomplete, and exogenous peptide delivery methods require optimization. Sustained research is therefore needed to identify novel targets and refine delivery strategies for bioactive peptides in neurodegenerative diseases (Wei et al. 2024).

#### 2.2.3 Neuropeptides

Neuropeptides are endogenous bioactive molecules that regulate neural signaling, with significant potential for AD prevention and treatment. Key examples include melatonin, apelin-13, and irisin. Apelin-13 and irisin mitigate apoptosis, oxidative stress, mitochondrial dysfunction, and neuroinflammation via specific pathways. Melatonin primarily inhibits the amyloidogenic processing of amyloid precursor protein (APP), reducing Aβ production (Wei et al. 2024).

Melatonin, an indole heterocycle, regulates circadian rhythms and induces endogenous antioxidant enzymes, exerting neuroprotection. Srivastava et al. (2020) developed a dopamine-melatonin nanocomposite (DM-NC) that inhibited Aβ nucleation, self-propagation, and fibril formation *in vitro*. Fihurka et al. (2023) designed a multitarget nanoagent (MIT) containing melatonin, insulin, and tetrahydrocannabinol. This formulation reduced tau phosphorylation and the expression of the mitochondrial dysfunction marker creatine kinase mitochondrial 1 (CKMT1) while improving spatial memory in AD mice.

Notwithstanding their promise, neuropeptides require further validation before clinical translation. Critical gaps include randomized clinical trials in AD patients or at-risk individuals, optimized dosing and administration schedules, and comprehensive characterization of long-term safety profiles (Zhang et al. 2025).

### 2.3 Systemic administration

Effective delivery of AD nanotherapeutics to brain neurons remains a major challenge owing to the complex CNS physiological microenvironment. Following systemic administration, nanodrugs are susceptible to enzymatic degradation, nonspecific tissue diffusion, and sequestration by endothelial barriers, the extracellular matrix and the BBB. The BBB acts as a key obstacle, reducing cerebral drug absorption, accelerating systemic excretion and preventing efficient neuronal accumulation. Additionally, the acidic intracellular microenvironment and lysosomal enzymes can further compromise nanodrug efficacy (Wang et al. 2021). The optimal *in vivo* delivery routes (injection, oral or nasal administration) are thus critical, as each dictates the biological barriers encountered, cerebral entry efficiency and systemic biodistribution profile. Nasal administration has emerged as a prominent strategy for AD nanotherapy due to its unique inherent advantages.

#### 2.3.1 Injection administration

Systemic injection enables direct delivery of nanodrugs into the circulatory system or CSF compartment, with each route exhibiting distinct BBB penetration mechanisms and inherent limitations.

Intravenous (IV) injection facilitates rapid systemic distribution and delivery of large nanodrug volumes, making it suitable for acute AD symptom management. IV-administered NPs cross the BBB primarily via receptor-mediated transcytosis or passive diffusion. For example, a Cy5.5-labeled ibuprofen and FK506-encapsulated codelivery system (Ibu&FK@RNPs) (1 mg·kg^−1^ Cy5.5-Ibu, equivalent to 50 mg·kg^−1^ RNPs) was intravenously administered to AD mice; *in vivo* imaging confirmed successful BBB penetration and cerebral accumulation. These NPs specifically bind to the receptor for advanced glycation end products (RAGE), which is overexpressed in the diseased AD neurovascular unit, mediating transcytosis and significantly enhancing brain accumulation relative to nontargeted NPs (He et al. 2022). IV injection also supports the delivery of high stem cell doses, a valuable strategy for AD regenerative therapy (Pires et al. 2022). However, Colby et al. (2023) reported critical limitations of IV delivery: NPs are rapidly cleared by the reticuloendothelial system (RES), resulting in low cerebral targeting efficiency (a median of 0.7% for most IV-administered nanosystems). Expansile NPs (eNPs) showed <2% tumoral accumulation in intraperitoneal mesothelioma models via IV injection compared with 65% via intraperitoneal administration, highlighting the profound impact of RES clearance on targeting efficacy. Nonspecific accumulation in healthy tissues additionally increases the risk of off-target toxic effects.

Intraperitoneal (IP) injection leverages the high absorptive capacity of the peritoneum, with some studies reporting drug absorption rates 3,000-fold faster than IV delivery (Wu et al. 2023). Saffari et al. (2020) demonstrated that metformin-loaded phosphatidylserine nanoliposomes (MET-PSLs) administered IP to AD-induced rats crossed the BBB via lipid-mediated transport, improving therapeutic bioavailability. At 22 days post-IP injection, MET-PSL significantly improved learning and memory indices (*p* < 0.05) and reduced hippocampal levels of tumor necrosis factor-alpha (TNF-α) and transforming growth factor-beta (TGF-β) in streptozotocin-induced AD rats (*p* < 0.05), confirming the feasibility of IP administration for AD nanotherapeutics. However, IP-delivered nanodrugs still require systemic circulation to reach the BBB, leading to partial RES clearance and reduced cerebral accumulation compared with direct CNS delivery routes.

Direct CSF injection (intracerebroventricular or intrathecal) completely bypasses the BBB, achieving high cerebral targeting specificity. For example, intracerebroventricular injection of miR-17 antagonist-loaded lipid NPs (anti-17 MLNPs) in 5xFAD mice enabled microglia-specific targeting with minimal off-target effects (Badr et al. 2024). Similarly, the hippocampal injection of gold NPs (AuNPs) directly delivered cargo to AD pathological lesions, evading systemic biological barriers (Sanati et al. 2019). Ralvenius et al. (2024) reported that intracisternal injection of anti-*PU.1* small interfering RNA (siRNA)-loaded NPs resulted in widespread cerebral distribution and reduced neuroinflammation in AD models, whereas IP injection caused nonspecific systemic organ distribution. Nevertheless, direct CSF injection is an invasive procedure requiring stereotaxic surgery, with associated risks of infection, local inflammation, and tissue damage. It also exhibits poor clinical translatability for long-term AD management due to significant patient compliance challenges.

In summary, injection-based delivery systems are hindered by inherent limitations. The IV and IP routes suffer from rapid RES clearance; Colby et al. (2023) performed a systematic review of 117 NP studies and reported a median IV delivery efficiency of 0.7%, underscoring this universal challenge. Direct CSF injection, while enabling complete BBB bypass, is clinically impractical for chronic AD therapy owing to its invasiveness. A clear trade-off also exists between invasiveness and efficacy: targeted NPs (e.g. Ibu&FK@RNPs (He et al. 2022)) show enhanced IV delivery via RAGE targeting, but their cerebral accumulation remains lower than that of intranasally or CSF-injected NPs. Higher IV NP doses may improve brain accumulation but increase toxicity risks, as exemplified by high-dose IV cerium oxide NPs (CNPs), which exhibit poor physicochemical stability and systemic side effects compared with low-dose intranasal administration (Danish et al. 2022). Collectively, injection route selection for AD nanotherapeutics must be tailored to the nanocarrier's pharmacokinetic profile, the target CNS drug concentration, and the clinical practicality of repeated administration in the aged AD population.

#### 2.3.2 Oral administration

IV formulations carry elevated risks of blood- and catheter-associated infections, whereas oral administration offers substantial clinical, resource, and cost benefits—including a reduced carbon footprint by eliminating IV equipment manufacturing. Oral formulations also align with patient preferences for chronic therapy, improving long-term adherence, a critical factor in AD management (Eii et al. 2023). Despite these advantages, brain-targeted oral drug delivery remains highly challenging, hindered by both the intestinal epithelial barrier (IEB) and the BBB.

To overcome the IEB, oral nanosystems are engineered with mucus-penetrating properties and ligand-mediated targeting capabilities. For example, mannose-modified PLGA-PEG NPs encapsulating fingolimod (FTY@Man NPs) bind intestinal epithelial receptors via mannose ligands, triggering transcytosis across both the IEB and the BBB. This dual-barrier penetration enabled cerebral accumulation of fingolimod and cognitive improvement in AD mice (Lei et al. 2024). Guo et al. (2023) demonstrated that oral chiral gold NPs (*l*-Au NPs) enhance BBB penetration by remodeling the gut‒brain axis, with indole-3-acetic acid (IAA) as the key mediator. The rigid structure of *l*-Au NPs resists gastric acid and proteolysis, allowing stable gastrointestinal transit to modulate the gut microbiome—specifically increasing the abundance of IAA-producing bacteria (e.g. *Clostridium*, *Lactobacillus*). This remodeling elevates intestinal IAA production, a metabolite deficient in the serum and CSF of AD patients. Cano et al. (2019) further validated that oral epigallocatechin gallate/ascorbic acid (EGCG/AA) NPs maintain stable plasma EGCG concentrations (~500 ng·mL^−1^ at 24 h), in contrast to free EGCG, whose levels decline rapidly (60.267 ± 40.150 ng·mL^−1^ at 24 h). Mechanistically, EGCG/AA NPs disrupt BBB tight junctions *in vitro* and *in vivo*, promoting EGCG cerebral penetration—an effect not observed with free EGCG. This finding indicates that nanocarrier-mediated modulation of barrier integrity may represent a viable strategy to enhance oral AD nanotherapy.

Oral nanosystems for AD face a cascade of translational barriers. Gastric acid, bile salts, and digestive enzymes readily degrade most oral nanosystems; unmodified lipid NPs, for instance, exhibit >50% degradation in simulated gastric fluid, leading to marked reductions in drug bioavailability (Liu et al. 2022). Even when nanosystems cross the IEB successfully, the BBB remains a formidable obstacle—most oral nanocarriers rely on indirect mechanisms (e.g. gut microbiota-derived metabolite production (Guo et al. 2023)) rather than direct BBB penetration, fundamentally limiting therapeutic efficacy. Additionally, ligand-modified oral NPs (e.g. mannose- or transferrin-functionalized) display highly variable BBB-targeting efficiency. Some studies report minimal cerebral accumulation, attributed to ligand degradation in the gastrointestinal tract or competitive inhibition by endogenous ligands at BBB receptor sites (Lei et al. 2024). The efficacy of such targeted systems thus depends on gastrointestinal ligand stability and high affinity for BBB receptors.

#### 2.3.3 Nasal administration

Nasal administration is a noninvasive route that bypasses the BBB via two primary pathways: olfactory nerve transcytosis and trigeminal nerve transport. This route evades systemic circulation, reduces RES clearance and gastrointestinal degradation, and confers superior brain targeting compared with injection and oral delivery routes.

Intranasal nanocarrier systems facilitate BBB penetration mainly by enhancing mucosal retention and promoting olfactory/trigeminal nerve-mediated transport. For example, intranasally administered CNPs exhibited 95.40 ± 0.006% free radical scavenging activity in brain tissue, alongside significant upregulation of superoxide dismutase and glutathione levels—effects attributed to direct nose-to-brain translocation (Danish et al. 2022). Additionally, intranasal delivery of CUR-loaded micellar emulsions (prepared with albumin NP precursors) achieved a brain CUR concentration of 141.5 ± 55.9 ng·g^−1^, whereas no CUR was detectable after intravenous administration of an equivalent dose. The resulting brain-to-plasma concentration ratio reached 7.06, markedly exceeding that of conventional micellar formulations (0.06), which confirms the superior brain-targeting efficiency of intranasal NP delivery (Sintov 2020).

To avoid gastric degradation and gastrointestinal adverse effects of oral administration, Georgieva et al. (2023) developed chitosan NPs encapsulating galantamine hydrobromide nanocrystals. The mucoadhesive properties of chitosan prolong the nasal residence time and enhance olfactory absorption, thereby facilitating direct CNS delivery. Liu et al. (2022) further demonstrated that intranasally administered mesoporous silica NP-coated *Bifidobacterium* (MSNs-Bi) can translocate via central pathways to the intestinal periphery and modulate short-chain fatty acid levels—a gut‒brain axis mechanism with potential therapeutic value for AD.

Advanced formulation strategies (e.g. microneedle and hydrogel-based systems) address intrinsic limitations of conventional nasal delivery, including mucociliary clearance and epithelial barrier resistance. Ruan et al. (2024) engineered a rapidly dissolving microneedle patch composed of hyaluronic acid and tannic acid-crosslinked gelatin, which releases cyclodextrin-based metal–organic frameworks (CD-MOFs) within seconds to enhance mucosal penetration. Similarly, hydrogel-assisted platforms, the black phosphorus (BP) and methylene blue (MB) composite hydrogel (BP-MB@Gel) and thermoresponsive hydrogel curcumin-loaded mesoporous silica nanoparticles (HG@MSN-CCM) improve mucosal retention and enable sustained, controlled drug release, thereby further enhancing BBB permeability (Ribeiro et al. 2022; Liu et al. 2024). These integrated systems consistently achieve superior brain accumulation compared with conventional nasal drops; For instance, NP-mediated rivastigmine transport across the nasal mucosa reached 73.3% efficiency versus 52% for free drug solution (Musumeci et al. 2022). Collectively, accumulating evidence confirms that intranasal administration optimizes nanomedicine brain entry via coordinated improvements in mucosal permeability, retention, and absorptive capacity (Yang et al. 2020; Yang et al. 2022; Islamie et al. 2023).

Nasal administration faces multiple interdependent barriers that collectively limit its translational potential for AD therapy. First, the nasal epithelium mediates rapid mucociliary clearance (10–20 min) and tight junction-restricted paracellular transport, leading to rapid elimination of unmodified NPs and necessitating mucoadhesive or permeation-enhancing surface modifications (Ruan et al. 2024). Second, the limited volumetric capacity of the human nasal cavity (15–20 μL per nostril) severely restricts the administrable absolute dose, which is a key challenge for chronic AD regimens requiring sustained therapeutic drug concentrations. Third, significant interindividual variability in nasal anatomical features (e.g. olfactory epithelial surface area, mucosal thickness, and ciliary function) results in inconsistent brain-targeting efficiency across patient populations; elderly AD patients, who often present with age-related nasal mucosal atrophy, may exhibit markedly reduced NP absorption (Georgieva et al. 2023).

#### 2.3.4 Critical knowledge gaps and unresolved translational challenges

Despite substantial progress in nanomedicine-enabled delivery strategies for AD research, critical knowledge gaps and unresolved challenges persist beyond generic technical barriers. These deficiencies impede clinical translation and limit therapeutic optimization, even for the most advanced nanocarrier platforms.

First, BBB penetration specificity remains suboptimal. Current nanosystems (e.g. RAGE-targeted nanosystems (He et al. 2022) and mannose-modified nanosystems (Lei et al. 2024)) lack precise targeting to AD pathological lesions (e.g. the hippocampus and cerebral cortex) and frequently exhibit off-target binding in healthy brain regions, reducing the therapeutic index. Second, rapid nanocarrier clearance or degradation compromises long-term therapeutic efficacy. Intravenous NPs typically have an *in vivo* half-life of <24 h (Colby et al. 2023), necessitating frequent dosing for sustained therapeutic effects. Additionally, stimulus-responsive nanosystem (e.g. ROS-responsive (He et al. 2022) and pH-responsive (Liu et al. 2024) formulations) require further optimization to achieve lesion-specific, spatiotemporally precise sustained drug release. Third, translational applicability is hindered by invasive delivery routes (e.g. direct CSF injection) and complex formulations (e.g. microneedles (Ruan et al. 2024). Scalable, low-cost nasal or oral nanosystems that retain therapeutic efficacy in human trials are therefore urgently needed. Fourth, safety and toxicity data remain incomplete. While NPs may induce nasal mucosal irritation (Georgieva et al. 2023) or RES overload (Colby et al. 2023), long-term preclinical evaluations of their biocompatibility and cumulative toxicity are lacking. Finally, barriers to combination therapy persist: co-loaded drugs often display differential BBB penetration efficiencies, and nanosystems enabling synchronized release of multiple synergistic cargos remain underdeveloped. Addressing these gaps demands interdisciplinary efforts to optimize targeting precision, prolong systemic circulation, simplify formulations and validate long-term safety—ultimately advancing AD nanomedicine from preclinical promise to clinical utility.

### 2.4 Penetration of the blood–brain barrier

After overcoming the physiological barriers, BBB penetration becomes the core bottleneck for treating CNS diseases. The BBB effectively isolates the CNS from blood-borne toxins and pathogens, yet it simultaneously hinders the delivery of most therapeutic agents (e.g. adeno-associated viruses and other gene delivery vectors) to the brain, which represents a fundamental bottleneck for most CNS-directed research tools and therapeutic strategies (Wu et al. 2023).

**Figure 3.**
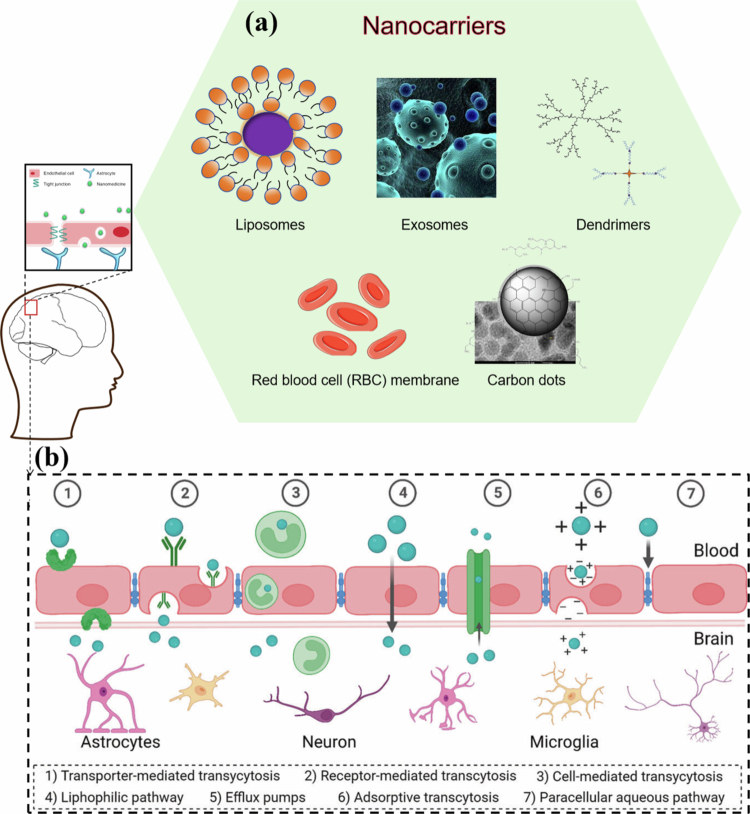
Nanocarrier platforms and BBB penetration pathways for central nervous system drug delivery. (a) Schematic representation of various nanocarriers engineered for BBB traversal, including liposomes, exosomes, dendrimers, red blood cell (RBC) membrane-coated carriers, and carbon dots. (b) Illustration of the major transport routes across the BBB: (1) transporter-mediated transcytosis, (2) receptor-mediated transcytosis, (3) cell-mediated transcytosis, (4) lipophilic pathway, (5) efflux pumps, (6) adsorptive transcytosis, and (7) paracellular aqueous pathway. These mechanisms enable nanocarriers to overcome the BBB and deliver therapeutics to the brain parenchyma, targeting neurons, astrocytes, and microglia. (a) Created with Bioicons.com and SciDraw.io; (b) Reprinted from Navarro Martínez et al. (2023) under a Creative Commons (CC BY 4.0) License.

To overcome this challenge, various engineered nanocarriers have been developed to increase BBB penetration. Drug molecules traverse the BBB via multiple pathways, including paracellular and transcellular diffusion, receptor-mediated transcytosis, cell-mediated transcytosis, transporter-mediated transcytosis, and adsorptive-mediated transcytosis (Figure 3b). For example, transcellular lipophilic diffusion permits the passage of small lipophilic molecules across the endothelial cell membrane, while cell-mediated transcytosis employs immune and stem cells (macrophages, stem cells, leukocytes) as ‘Trojan horses’ for drug delivery (Navarro Martínez et al. 2023). By tailoring their physicochemical properties (e.g. size, surface charge, and ligand decoration), NPs can exploit one or more of these routes. Figure 3a illustrates the major nanocarrier platforms engineered for BBB traversal: liposomes, exosomes, dendrimers, red blood cell (RBC) membrane‑camouflaged, and CDs (Aliev et al. 2019; Duan et al. 2023; Naimi et al. 2024). Inorganic nanocarriers (MNPs, Au NPs) are discussed in the clinical diagnosis section.

#### 2.4.1 Liposomes

Liposomes represent a widely investigated nanocarrier platform for brain delivery, particularly for therapeutic agents unable to cross the BBB independently (Hernandez and Shukla 2022). Their translational potential arises from their excellent biocompatibility and, more importantly, facile surface functionalization with ligands that exploit receptor-mediated transcytosis—a key pathway for BBB penetration (Song et al. 2025).

For instance, lysophosphatidylcholine is directly integrated into liposomes through hydrophobic interactions between its acyl tail and the liposomal lipid bilayer. This modification not only preserves the drug-loading capacity of liposomes but also endows them with the unique function of activating brain endothelial transcytosis. Consequently, this engineered liposomal system exhibits a 2.3-fold higher brain drug accumulation compared to conventional liposomes (Zhan et al. 2026). Gu et al. (2024) fabricated PEGylated liposomes encapsulating astaxanthin (PEG-ATX@NPs) with a sub-100 nm hydrodynamic diameter to enhance brain entry. In a separate approach, CUR nanoparticle-loaded cardiolipin liposomes (RCLs@CNPs) were engineered to protect felodipine from systemic clearance, sustaining adequate drug concentrations at the BBB interface during barrier opening (Feng et al. 2024).

Despite these advances, the clinical translation of liposomal nanocarriers remains limited by formulation- and platform-specific safety challenges (Wang et al. 2024). Cationic liposomes have high drug delivery efficiency; however, their positively charged surfaces often induce cytotoxicity, with cell mortality reaching up to 45%. This effect is attributed to unfavorable electrostatic interactions between lipid vesicles and negatively charged serum macromolecules. This limitation has been partially alleviated using PEGylated neutral lipid formulations (e.g. 1,2-distearoyl-sn-glycero-3-phosphocholine:cholesterol:2-distearoyl-sn-glycero-3-phosphoethanolamine-N-[amino(polyethylene glycol)-2000]) (Andrade et al. 2022).

Nonetheless, further optimization is needed for such lipid components in brain-targeted formulations to preserve colloidal stability and ligand functionality in the CNS microenvironment.

#### 2.4.2 Exosomes

Exosomes are nanoscale, membrane-bound extracellular vesicles secreted by diverse cell types. Owing to their endocytic origin, they intrinsically express surface proteins such as tetraspanins (CD9, CD63, and CD81) and integrins, which facilitate receptor-mediated transcytosis across brain endothelial cells, conferring natural BBB-traversing ability (Rehman et al. 2023; Mehdizadeh et al. 2025). The nanoscale dimensions and lipid bilayer structure of these peptides also enable passive diffusion and protect encapsulated payloads from enzymatic degradation in the circulation (Mehdizadeh et al. 2025).

Surface engineering with brain-homing peptides further enhances exosomal BBB penetration. For instance, the core mechanism underlying BBB penetration by multitarget engineered exosomes involves a hybrid exosome membrane constructed by fusing brain microvascular endothelial cell-derived exosomes (bEnd.3 Exo) and macrophage-derived exosomes (RAW264.7 Exo) (Du et al. 2025). This membrane hybridization strategy inherits the intrinsic brain-homing capacity of bEnd.3 Exo, which are secreted by BBB-resident endothelial cells and display natural affinity toward the BBB microenvironment. Jiang et al. (2024) functionalized exosomes with angiopoietin-2 (Ang2) polypeptides to enhance transcytosis via receptor-mediated pathways, achieving preferential accumulation in AD-affected brain regions. Similarly, Li et al. (2023) conjugated rabies virus glycoprotein to mesenchymal stem cell-derived exosomes, enabling siRNA delivery across the BBB through targeted receptor recognition.

Despite these advantages, the clinical translation of exosome-based nanocarriers is hampered by platform-specific limitations. The main feature among these is inherent heterogeneity: exosomes isolated even from clonal cell lines display wide variations in size (30–150 nm), surface marker density, and cargo composition, which directly compromise batch consistency and therapeutic predictability (Mehdizadeh et al. 2025). Unlike synthetic carriers, exosomes lack standardized isolation and purification protocols; current methods (ultracentrifugation, size-exclusion chromatography) are labor-intensive, low-yield, and are difficult to scale under good manufacturing practice (GMP) conditions (Li et al. 2024). Although bioreactor-based production systems show promise, cost-effective, large-scale manufacturing with reproducible quality remains an unmet need. Furthermore, the regulatory classification of exosome therapeutics remains ambiguous—whether as biologics, drugs, or devices—creating uncertainty in approval pathways and delaying clinical translation (Mehdizadeh et al. 2025).

#### 2.4.3 Dendrimers

Dendrimers are branched, monodisperse polymers (e.g. polyamidoamine [PAMAM], polypropylene imine [PPI], PLGA, PEG) with well-defined nanoscale architecture and densely functionalizable surfaces (Beg et al. 2011). Their BBB traversal is primarily mediated by the multivalent effect: multiple surface ligands simultaneously engage endothelial receptors, enhancing binding avidity and triggering receptor-mediated transcytosis (Beg et al. 2011; Romero-Ben et al. 2025). This polyvalent interaction distinguishes dendrimers from linear polymers or liposomes, enabling efficient brain entry even at low doses.

Surface engineering further optimizes their BBB penetration: for example, the maltose–histidine shelling of PPI dendrimers (G4HisMal) promotes adsorptive-mediated transcytosis via electrostatic interactions with the negatively charged endothelial glycocalyx (Aso et al. 2019). The conjugation of vitamin A and tocopheryl polyethylene glycol succinate-1000 to PAMAM scaffolds facilitates receptor-targeted brain delivery, though their endocytic pathways remain incompletely elucidated (Singh et al. 2022). Beyond drug delivery, the high surface area of dendrimers supports diagnostic applications: gold nanoparticles–PAMAM nanocomposites (Au‑PAMAM) immobilized on electrodes enable ultrasensitive tau protein detection (limit of detection of 1.7 pg·mL^−1^) in plasma and brain tissue, leveraging multivalent signal amplification to distinguish AD patients from healthy controls (Razzino et al. 2020).

Despite structural precision, dendrimers face unique physicochemical constraints that impede clinical translation (Romero-Ben et al. 2025). Polyester-based dendrimers (e.g. PLGA) generate acidic degradation byproducts (lactic and glycolic acid), lowering the local pH, perturbing the brain microenvironment, and destabilizing coencapsulated biologics. Most polyester backbones also lack pendant reactive groups, severely limiting postsynthetic functionalization without elaborate copolymerization. Additionally, the organic solvents and high shear stress required for dendrimer‒drug conjugation/encapsulation can denature sensitive biomacromolecular cargoes (e.g. antibodies and enzymes). These platform-specific limitations are the primary translational barriers for dendrimer-based AD therapeutics.

#### 2.4.4 RBC membrane or other cell membrane camouflage

RBC membrane-camouflaged nanocarriers (RBCm-nanocarriers) adopt a biologically inspired strategy to traverse the BBB. Coating synthetic NPs with erythrocyte membranes preserves core physicochemical properties (e.g. controlled drug release, hydrophobic payload accommodation) while conferring intrinsic biological functionalities of native red blood cells (Chai et al. 2019). A key feature is immune evasion: the transmembrane protein CD47 on the RBC membrane binds to signal regulatory protein-α on macrophages, inhibiting phagocytosis and significantly prolonging systemic circulation (Aryal et al. 2013). This extended bloodstream residence enhances passive BBB targeting, and the RBCm platform readily allows secondary surface modifications for active brain delivery.

Various engineering strategies have been developed to enhance BBB penetration. Liu et al. (2024) encapsulated carbon quantum dots and polydopamine in RBC membranes; the resulting nanocomposites used an immune-evasive RBC coating to facilitate brain accumulation and inhibit copper-mediated Aβ_42_ aggregation. Su et al. (2023) engineered a transferrin receptor aptamer-modified RBCm-nanodrug (TR-ZRA) that specifically recognizes brain endothelial transferrin receptors, enabling transcytosis into the brain parenchyma. Lin et al. (2024) developed a hybrid membrane strategy by fusing platelet membranes with chemokine (C-C motif) receptor 2 (CCR2)-overexpressing cell membranes. This coating utilized the high affinity of CCR2 for CCL2 (upregulated at neuroinflammatory sites) to achieve selective BBB penetration and lesion-targeted localization.

Despite these advances, RBCm-nanocarrier clinical translation is hindered by inherent manufacturing and quality control challenges (Yuan et al. 2025). Fabrication remains limited to laboratory-scale extrusion or sonication, with coating efficiency showing substantial batch-to-batch variability owing to donor-dependent differences in RBC membrane composition and CD47 expression. Unlike fully synthetic nanocarriers, RBCm-nanocarriers lack validated, scalable production workflows; existing methods are labor intensive, poorly reproducible, and incompatible with current GMP standards. Additionally, the biological origin of the membrane coating introduces intrinsic heterogeneity, complicating characterization, and definition of critical quality attributes (CQAs)—including membrane protein integrity, surface ligand density, and vesicle size distribution—thus impeding robust release specification establishment and streamlined regulatory approval.

#### 2.4.5 Carbon dots (CDs)

CDs are zero-dimensional nanomaterials composed of graphitized carbon cores passivated with polymeric surface groups, featuring ultrasmall dimensions (2–10 nm), intrinsic photoluminescence, high photostability, and favorable biocompatibility (Wang et al. 2022). Their small size enables passive BBB traversal via the fenestrated endothelium or transcellular diffusion—distinct from the receptor- or transporter-mediated transcytosis of larger nanocarriers. The abundant carboxyl and amine moieties on the CD surfaces allow facile conjugation with brain-targeting ligands (e.g. transferrin), facilitating active transcytosis across brain capillary endothelial cells. Quantitative ¹³C-labeling studies show CDs brain parenchyma accumulation is three-fold higher than that of conventional polymeric NPs or liposomes, highlighting superior BBB penetration efficiency (Sethumadhavan et al. 2025).

Various CD engineering strategies have been developed to enhance brain entry. Zhou et al. (2019) synthesized amphiphilic yellow-luminescent CDs (y-CDs) (with a mean diameter of 3 nm) via ultrasound-mediated condensation of citric acid and o-phenylenediamine. These particles had high surface densities of primary amine (6.12 × 10⁻⁵ mmol·mg^−1^) and carboxyl (8.13 × 10⁻³ mmol·mg^−1^) groups, facilitating bioconjugation and passive BBB diffusion without disrupting tight junction integrity. Zhang et al. (2024) derived congo red-functionalized CDs (CRCDs) from citric acid and Congo red; CRCD1 exhibited strong BBB traversal capacity and dual targeting of Aβ₄₂ and tau aggregates, as evidenced by low IC₅₀ values (2.1 ± 0.5 and 0.2 ± 0.1 μg·mL^−1^, respectively). Kuang et al. (2020) fabricated Fe₃O₄@CD composites, which enhanced CUR cellular uptake via CD-mediated endocytosis, demonstrating the versatility of CDs as brain delivery vectors.

Despite promising CNS drug delivery potential, CDs face significant translational barriers (Wang et al. 2022; Zhang et al. 2025). Long-term *in vivo* toxicity and biodistribution are poorly characterized; their chemically stable carbon core raises concern over biodegradability and chronic accumulation in the brain or RES. Bottom-up synthesis introduces substantial batch-to-batch variability, hindering pharmaceutical-grade manufacturing standards. Additionally, the lack of regulatory precedent and consensus on CNS-targeted CD CQAs exacerbates translational challenges. Overcoming these challenges requires integrated advances in nanotoxicology, process engineering, and AI-assisted design to enable scalable production of clinically viable CD-based theranostics.

#### 2.4.6 Comparative evaluation of brain-targeting nanocarriers: controversies and translational challenges

The preceding sections detail the unique advantages and challenges of individual nanocarrier platforms. To facilitate a clear translational perspective, a direct comparison across the critical axes of BBB penetration efficiency, safety profile, scalability, and clinical translatability is imperative. Table 2 provides a summarized cross-comparison of liposomes, exosomes, dendrimers, RBCm carriers, and CDs, including their characteristic size ranges.

**Table 2.** Comparative analysis of nanocarriers for AD therapeutics.

Nanocarrier	Characteristic size ranges	BBB penetration efficiency	Safety	Scalability	Clinical translatability
Liposomes	50–200 nm	Moderate-high; receptor-mediated transcytosis	Moderate-high; surface modification-dependent	Moderate; cationic types with cytotoxicity risk	High; mature processes for large-scale production
Exosomes	30–150 nm (~100 nm avg)	High; endogenous transcytosis; peptide ligand potentiation	High; innate ability, cell-source-dependent	High; biocompatible, low immunogenicity	Low-moderate; low yield, batch variability
Dendrimers	1–10 nm (G3–G7)	Moderate; multivalent effect-mediated uptake; tunable size/surface functionalit	Moderate; generation/modification-dependent	Moderate; acidic degradation may disrupt microenvironment	Low-moderate; tedious synthesis, poor monodispersity
RBCm-Carriers	50–200 nm	Moderate-high; receptor-mediated transcytosis	High; CD47-mediated immune evasion enhances crossing	High; natural tolerance, low toxicity/inflammation	Low-moderate; RBC source-dependent, batch instability
CDs	1–10 nm	High; endogenous transcytosis; peptide ligand potentiation	Moderate; size-facilitated diffusion, ligand-dependent targeting	Moderate; long-term accumulation toxicity unconfirmed	High; ‘bottom-up’ synthesis for scaling

The comparative analysis in Table 2 reveals a recurrent translational trilemma among high penetration efficiency, excellent safety, and scalable manufacturability. For instance, while exosomes and RBCm carriers leverage biological mechanisms (e.g. CD47-mediated immune evasion for RBCm carriers) for superior BBB crossing and biocompatibility, their inherent biological complexity severely compromises production scalability and batch uniformity, which are critical parameters for regulatory approval. Conversely, synthetic platforms such as liposomes and dendrimers offer greater compositional control and have established regulatory pathways; however, their brain delivery efficiency often relies on complex surface engineering and can be unpredictable *in vivo*. For instance, the positive surface charge of traditional cationic liposomes can induce cytotoxicity, while the acidic degradation byproducts of certain dendrimers may disrupt the local physiological microenvironment. Compared to liposomes, dendrimers generally exhibit lower targeting efficiency and payload capacity and are more challenging to synthesize with a monodisperse size distribution. Furthermore, issues such as sterilization difficulties present additional hurdles for their large-scale production compared to other nanocarrier platforms. In contrast, the synthesis of CDs, particularly via ‘bottom-up’ approaches (e.g. hydrothermal carbonization), is generally straightforward and well established (Yang 2025; Zhang et al. 2025). However, the stability conferred by their carbon-based core raises considerations regarding potential long-term *in vivo* toxicity, a subject of ongoing investigation (Begines et al. 2020).

A key convergent challenge across all platforms is the scalability–clinical translation gap. Most reviewed systems are confined to laboratory-scale proof-of-concept studies. Transitioning to robust, cost-effective, and reproducible GMP production represents the most significant non-scientific hurdle. This is particularly acute for biologically sourced carriers (exosomes, RBCm-carriers) due to donor/batch variability but remains a substantial challenge even for synthetic nanocarriers (liposomes, dendrimers, CDs), where precise control over CQAs and stability must be maintained at scale. For dendrimers and liposomes, opsonization, and protein corona formation in the bloodstream result in rapid clearance by the mononuclear phagocytic system and a consequent reduction in therapeutic half-life (Li et al. 2021). Therefore, overcoming the current translation barriers will require a multidisciplinary convergence of materials science, process engineering, and rigorous translational neuropharmacology to tailor these sophisticated nanoplatforms for the clinical demands of AD therapy.

### 2.5 Stimuli-responsive drug release

The controlled-release mechanism of a drug—triggered after crossing the BBB and reaching target sites—is critical for therapeutic efficacy. Drug delivery systems are active therapeutic strategies that respond to exogenous or endogenous stimuli for localized release, transporting drugs to specific action sites. These systems utilize external physicochemical stimuli (e.g. NIR, ultrasound, and external ROS) and endogenous stimuli (e.g. pH responsiveness and enzymatic cleavage) (Ding et al. 2023; Liu et al. 2024). Controlled, sustained drug delivery reduces or eliminates side effects associated with high plasma drug concentrations or ‘dose dumping,’ while enabling better therapeutic concentration management, sustained blood levels, and prolonged efficacy (Anusha et al. 2023). However, developing precise nanomedicines that guide active ingredients to target regions via nanostructures, achieving drug release and enrichment, remains a key clinical challenge. Researchers continue to explore intelligent, efficient, controllable, and biocompatible responsive strategies.

#### 2.5.1 Near-infrared light (NIR) response

Traditional visible light (e.g. blue, yellow light) has shallow tissue penetration and may induce side effects such as tissue damage and unnecessary inflammation. In contrast, emerging deep-penetrating NIR light has attracted significant attention due to its relative biocompatibility. NIR-activated optogenetic tools provide a less invasive approach for remote control applications (Yu et al. 2019). Previous studies have demonstrated that NIR light inhibits neuroinflammation in neurodegenerative diseases, including PD, AD, amyotrophic lateral sclerosis (ALS), and atherosclerosis (Chai et al. 2024; Liu et al. 2024). Given the high sensitivity of Aβ fibrils to pH and temperature and their decreased stability with increasing temperature, photothermal therapy is a potential strategy for decomposing Aβ fibrils (Liu et al. 2023).

The photothermal effect elicited by NIR light combats AD through a dual mechanism. First, localized photothermal effects can transiently disrupt the BBB, facilitating transcytosis of therapeutic agents across this physiological barrier. Second, the photothermal response enables efficient dissociation of Aβ aggregates and hyperphosphorylated tau protein, while suppressing de novo Aβ aggregation. For example, Ye et al. (2023) and Ge et al. (2022) explored combining nanomedicines with NIR photocatalysis and photothermal therapy for AD treatment. Their research showed that under NIR irradiation, nanocomposites not only enhanced catalytic performance and photothermal conversion efficiency but also significantly improved BBB permeability via strong photothermal effects. These systems further responded to lesion-specific phase transitions to treat AD.

#### 2.5.2 Ultrasonic response

Sonodynamic therapy (SDT) employs ultrasound at specific intensities and frequencies generally considered safe for biological tissues, triggering microbubble resonance via cavitation or sonoporation effects. Specifically, when a microgas core is enclosed in a unidirectional open space, stimulation at its resonant frequency generates a driving force at the gas‒liquid interface, enabling ultrasound-controlled directional microcarrier movement. SDT has broad applications (e.g. biosensing, diagnostic imaging, treatment of various diseases) due to the diverse range of substances responsive to ultrasound (Huang et al. 2023).

For example, Ma et al. (2020) used fluorocarbon cores encapsulated in inorganic compound liposomes; these cores undergo phase transition to form microbubbles upon heating, serving as contrast agents for enhanced ultrasound diagnosis. For ultrasound-triggered therapeutic applications, Dong et al. (2021) employed bismuth molybdate (BMO) nanoribbons for efficient SDT-based antitumor therapy. Notably, ultrasound-sensitive coated microbubbles formulated in targeted lipid nanoemulsions offer a promising platform for AD treatment. Incorporating therapeutic agents into targeted lipid-soluble microbubble (LCM) or nanoparticle-derived (ND) nanoemulsions enables simultaneous, localized drug delivery across diverse AD-relevant cell types (D'Arrigo 2018).

Specifically, for AD therapy, Liu et al. (2020) encapsulated QUE-modified sulfur nanoparticles (QUE@SNPs) within microbubbles (MBs). This system leverages focused ultrasound (FUS)-mediated transient BBB opening to facilitate the accumulation of QUE@SNPs in the brain parenchyma of AD mice. Similarly, Deng et al. (2021) demonstrated that microbubble-enhanced FUS (MB-FUS) noninvasively opens the BBB, facilitating the brain-targeted delivery of ultrasound-stimulated exosomes derived from human astrocytes (US-HA-Exo). Ultrasound stimulation increased exosome release from HA cells by nearly fivefold compared to untreated controls; critically, these delivered exosomes effectively cleared Aβ plaques and reduced Aβ-induced neurotoxicity.

#### 2.5.3 Reactive oxygen species (ROS) response

Reactive oxygen species (ROS) are diverse molecular oxygen derivatives generated during normal aerobic metabolism that act as key signaling molecules. They modulate signal transduction pathways by directly reacting with proteins, transcription factors, and genes, inducing functional alterations via structural modifications. Hydrogen peroxide (H₂O₂), a nonradical ROS, can reversibly oxidize critical redox-sensitive cysteine residues on target proteins. Mild ROS elevation induces transient cellular alterations that stimulate cytokine production and mediate inflammation, while severe increases cause irreversible oxidative damage and cell death (Trachootham et al. 2009). Elevated ROS levels are strongly associated with pathologies such as cancer and inflammation, enabling therapeutic exploitation via responsive nanomedicines.

Zhang et al. (2023) demonstrated that abundant ROS trigger the responsive release of puerarin from nanodrugs, repairing mitochondrial function by maintaining ATP metabolism and membrane integrity. This inhibits cytochrome C-mediated apoptosis, showing potential for ischemic stroke treatment. Similarly, Li et al. (2024) found that nanoencapsulated supramolecular drugs respond to and scavenge high ROS levels at cardiovascular injury sites, alleviating endoplasmic reticulum stress-related pathologies. Oxidative stress and neuroinflammation are well-established contributors to AD pathogenesis, with AD lesion sites characterized by high ROS concentrations. Capitalizing on this feature, researchers have designed ROS-responsive drug delivery systems for AD. Yang et al. (2023) designed NP systems that rapidly release nicotinamide adenine dinucleotide (NAD^+^) and Beclin1 (a mitophagy promoter) in high-ROS environments. This restores mitochondrial homeostasis and polarizes microglia to the M2 phenotype, enabling Aβ phagocytosis. In response to H₂O₂, Qiao et al. (2023) created UCNPs@mSiO₂-MB@AuNPs (USMA), a multifunctional nanocomposite. H₂O₂ cleaves USMA's boronate ester bonds, detaching AuNPs and releasing MB from mesoporous silica; AuNPs inhibit Aβ aggregation, while MB suppresses tau aggregation.

#### 2.5.4 pH responsive

pH-responsive polymers (e.g. certain PEG derivatives) contain ionizable acidic or basic residues whose ionization state depends on the solution pH. These materials undergo specific physicochemical changes (e.g. charge alteration) via proton acceptance or release in response to environmental pH (Tao et al. 2019). As a predominant stimulus-responsive mechanism in nanomedicine, pH sensitivity exploits key microenvironmental differences: tumor tissues have a lower pH (~6.5–7.0) than physiological blood (~7.4), while lysosomes have an acidic interior (pH ~4.5–5.5).

Du et al. (2018) leveraged this property by PEGylating NPs to evade rapid clearance, with acid-triggered PEG cleavage enabling targeted drug release in tumors—a strategy with significant potential for AD treatment. For brain delivery, Cai et al. (2020) designed transferrin receptor-targeted dendritic polylysine conjugated with acid-cleavable PEG. Lysosomal acidity triggers PEG dissociation, facilitating escape via charge reversal and enhancing T7 peptide internalization. Similarly, Qian et al. (2022) employed citrate-modified NPs, whose pH sensitivity enables lysosomal escape via charge switching. This platform delivered the HNSS/SS31 hybrid peptide to mitochondria, increasing its mitochondrial accumulation by 4.8-fold, which alleviated memory deficits and cholinergic neuron damage in 3xTg-AD mice. Alternatively, Wang et al. (2023) developed a calcium folate (CaFO) nanocomposite stable at neutral pH but degradable under lysosomal acidity, releasing calcium ions to accelerate neural stem cell differentiation and folate to direct cholinergic neuron specification—supporting stable acetylcholine (ACh) production, storage, and release.

#### 2.5.5 Biological enzyme response

Biological enzymes are environmentally friendly, nontoxic biocatalysts produced by living cells that accelerate biochemical reactions (e.g. protein-mediated processes, selected RNA functions). They exhibit strict substrate specificity and high catalytic selectivity (Vellard 2003). Compared to other stimuli-responsive nanomedicines, enzyme-responsive systems have intrinsic advantages: as endogenous substances, they require no external energy sources (e.g. ultrasound, light), ensuring biocompatibility and biosafety. Additionally, enzymatic reactions have rapid kinetics and precise substrate targeting, minimizing off-site drug release (Hu et al. 2012).

For example, Schiffmann et al. (2023) developed peptide‒polymer nanosystems carrying fluorescent labels and dexamethasone that identify sites of elevated matrix metalloproteinase expression in diabetic complications. MMP cleavage of hydrophilic peptide headgroups triggers an NP phase transition from the nanoscale to the microscale, enabling active retention in inflamed tissues. Similarly, Wang et al. (2022) leveraged differences between bacterial and mammalian thioredoxin systems to design thiol-targeted nanoinhibitors based on enzyme-reactive covalent organic frameworks (COFs), achieving precise ethaselen and Ag⁺ release at infection sites for anti-inflammatory effects and accelerated wound healing. These examples underscore a common theme: leveraging disease-associated enzymatic dysregulation—whether MMP upregulation in inflammation or pathogen-specific thioredoxin systems in infection—for targeted therapeutic intervention. This principle extends effectively to AD, where senescence-associated enzymes are similarly upregulated in affected brain regions. Ji et al. (2024) established significant correlations between β-galactosidase (β-gal) levels and AD-related genes, exploiting the fact that β-gal is markedly upregulated in senescent cells within the AD brain. They developed senolysis-specific killer 1 (SSK1)-NPs to deliver SSK1—a prodrug activated specifically by lysosomal β-gal—to affected brain regions. β-gal cleavage releases the active compound, which modulates senescence-associated gene expression, reduces Aβ burden, and attenuates cognitive deficits in aged mouse models.

#### 2.5.6 Ultra-short pulse electric field response

In recent years, ultrashort pulsed electric fields (PEFs)—characterized by high amplitude (several kV·m^−1^ to hundreds of kV·m^−1^) and short duration (milliseconds to microseconds)—have emerged as a potent physical agent capable of inducing reversible or irreversible cellular electroporation. Studies have demonstrated that PEF is a novel treatment for glioblastoma multiforme (GBM), with efficacy linked to transient BBB disruption and reduced tumor compactness, facilitating therapeutic agent penetration and enhancing drug delivery in immunotherapy and chemotherapy. The nonthermal nature of these electrical signals protects surrounding healthy tissues, enabling responsive treatment of localized masses, including those in sensitive anatomical regions (Casciati et al. 2022).

Growing interest in biocompatible nanotechnology combined with electric field stimulation has advanced the development of electro-sensitive smart drug delivery systems. Common electrosensitive materials include phospholipids, which can design nanoscale vesicles responsive to externally applied electric fields. For example, Qian et al. (2025) controlled liposomal drug release using nanosecond pulsed electric fields, employing aqueous fluorescent NPs to simulate anticancer drugs or therapeutic antibodies. Their study showed that ultrashort pulsed electric fields disrupted intercellular junctions, reduced the expression of adherens junction protein (N-cadherin) and tight junction protein (zonula occludens-1), and decreased adhesion while inhibiting epithelial‒mesenchymal transition. Under fluorescence microscopy monitoring, NP penetration into spheroids was positively correlated with the number of ultrashort pulsed electric field pulses, with higher fluorescence signals in the inner quiescent zone or necrotic core. These findings indicate that ultrashort pulsed electric fields combined with nanocarriers hold promise for targeted responsive treatment of diseases such as AD and, as an emerging technology for neurodegenerative disorders, warrant further in-depth investigation.

## 3 Mechanisms of nanomedicine in AD targeted therapy

As mentioned previously, chemically engineered NP surfaces increase BBB permeability, facilitating the targeted delivery of therapeutic agents to the brain. These nanocarriers achieve stimuli-responsive drug release at specific sites for AD intervention. The success of this delivery strategy has laid the foundation for subsequent pathological targeted therapy. The following text will further analyze how nanomedicines exert neuroprotection by regulating mechanisms such as Aβ aggregation.

**Figure 4.**
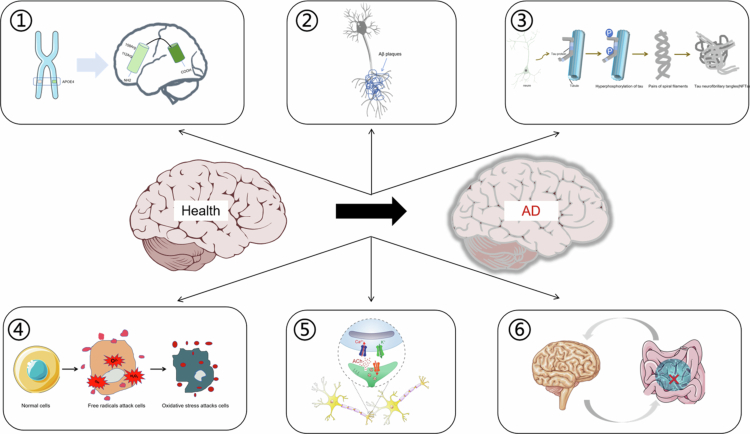
Convergence of major pathological hypotheses in AD pathogenesis. Schematic integration of the core mechanisms proposed to drive AD neuropathology: ① Genetic mutations (*APP*, *PSEN1/2*, *APOEε4*); ② Aβ pathogenic cascade; ③ tau hyperphosphorylation and neurofibrillary tangle formation; ④ oxidative stress mediated by free radical attack; ⑤ cholinergic injury due to impaired ACh synthesis and release; and ⑥ gut microbiota dysbiosis and associated metabolic influences. These interconnected hypotheses collectively contribute to the transformation of normal neurons into AD pathology, forming a synergistic network in which nanomedicine strategies target therapeutic intervention. Created with Bioicons.com and SciDraw.io.

**Figure 5.**
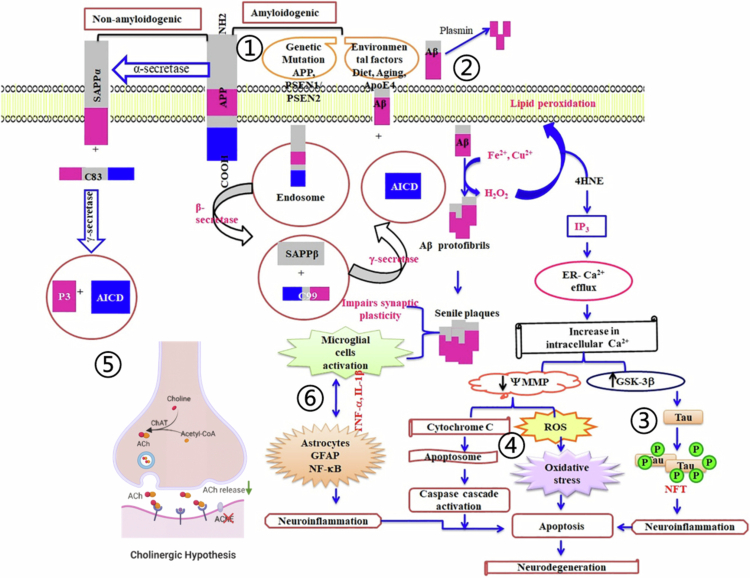
Molecular pathways linking AD risk factors to pathogenesis. Schematic diagram illustrating the processing of APP via nonamyloidogenic (α-secretase) and amyloidogenic (β- and γ-secretase) pathways, leading to Aβ aggregation and proteofibril formation. Aβ accumulation triggers microglial activation, neuroinflammation (via cytokines IL-1, IL-6, TNF-α, and NF-κB), ROS, and mitochondrial dysfunction (cytochrome C release, apoptosome formation, and the caspase cascade). Concurrently, tau hyperphosphorylation (indicated by multiple ‘P’ sites) leads to NFT formation. The cholinergic hypothesis is represented by ACh synthesis and release disruption. Numbered elements (①–⑥) correspond to the hypotheses and pathways overviewed in Figure 4. Reprinted from Asim et al. (2025) under a Creative Commons (CC BY 4.0) License; reprinted from Kesika et al. (2021) with permission from Elsevier, copyright 2021.

Advances in understanding AD pathology and related pathways have promoted the development of nanomedicine strategies. These strategies target key pathogenic mechanisms, as illustrated in Figures 4 and 5. Major approaches focus on regulating metabolic pathways to repair critical gene mutations, inhibiting Aβ and tau aggregation, suppressing neuroinflammation, and reducing oxidative stress and associated radical damage. Additional supportive strategies aim to modulate ACh levels, adjust the gut microbiota composition, and restore mitochondrial function (Asim et al. 2025). With ongoing research, emerging areas are also being explored, including the modulation of estrogen deficiency, which is considered to influence AD pathogenesis. Additionally, the pathways involved include two main categories: mutations in genes encoding *APP* and *PSEN1/PSEN2*, and inflammation-associated metabolic pathways. For example, Aβ plaque-activated microglia release cytokines and chemokines. These factors in turn activate astrocytes to secrete cytokines, chemokines, and acute-phase proteins, which further activate microglia (Kesika et al. 2021).

Owing to the multifactorial pathogenesis of AD, researchers have devoted substantial efforts to developing multifunctional nanosystems to increase treatment specificity and efficacy while minimizing side effects (Lo et al. 2025; Zhao et al. 2025). However, it is important to note that while the conceptual framework is robust, the vast majority of these strategies remain at the proof-of-concept or preclinical validation stage. The following sections detail these hypotheses and corresponding nanotherapeutic strategies, emphasizing their mechanistic rationale and contextualizing their current development stage.

### 3.1 Nanoparticles-enabled RNAi and gene therapy targeting AD-associated genes

#### 3.1.1 Gene mutation hypothesis

Early studies indicated that AD manifests in two forms: familial AD and sporadic AD. Mutations in *APP*, *PSEN1*, and *PSEN2* genes are strongly associated with FAD pathogenesis. In contrast, sporadic AD is primarily linked to the *APOE* gene on chromosome 19, where the *APOEε4* allele confers significantly increased susceptibility. Furthermore, genome-wide association studies have identified more than 20 genes linked to sporadic AD, including the chromosome 11-located *BACE1* gene—implicated in Aβ accumulation—and the R47H variant of the *TREM2* gene, which elevates AD risk. Collectively, these genetic alterations play a significant role in AD initiation and progression (Goate et al. 1991; Jonsson et al. 2013; Qin et al. 2016).

#### 3.1.2 Nanomedicine treatment strategies

Toxic amyloid aggregation, a key pathogenic event in AD, is mediated by *BACE1*, which processes APP. Consequently, *BACE1* silencing via siRNA represents a promising AD therapeutic strategy. However, effective brain delivery of siRNA remains challenging.

To overcome this limitation, Zhou et al. (2020) developed the Gal-NP@siRNA nanosystem, which exhibits superior blood stability and efficiently penetrates the BBB via glucose transporter 1 (GLUT1)-mediated transport. This approach delivered siBACE1, which significantly reduced the expression of BACE1, leading to a decrease in Aβ plaque levels, while also inhibiting the level of p-tau protein and regenerating damaged myelin basic protein. Notably, Gal-NP@siBACE1 administration reversed cognitive deficits in AD mice without significant side effects, demonstrating the utility of this ‘Trojan horse’ strategy for RNA interference therapy in neurodegenerative diseases. Similarly, an engineered nanosystem, designed to deliver siBACE1 across the BBB, was also shown to inhibit BACE1 expression and reduce Aβ plaque formation in *APP/PS1* mice. Furthermore, BACE-1 inhibition in microglia was accompanied by an upregulation of transcription factors (e.g. *Jun*, *Jund*, *Btg2*, *Fos*, *Fosb*), promoting a shift toward a damage-associated macrophage phenotype (Jiang et al. 2024).

In another preclinical study, Xu and his colleagues (Xu et al. 2025) used lactoferrin (Lf) to functionalize lipid nanoparticles (LNPs) and co-package α-mannosidase (α-M) and *BACE1* siRNA (siB) (α-M/siB@L-Lf) for nasal administration. The delivered siB showed significant BACE1 downregulation efficiency and delayed Aβ oligomer-induced pathological deterioration, indicating a significant neuroprotective effect (95.59%). This finding demonstrates the potential of nose-to-brain delivery of targeted lipid NPs as a two-pronged Aβ nanoscavenger for AD therapy.

### 3.2 Engineered nanoplatforms disrupting the Aβ pathogenic cascade: inhibition, disaggregation, and clearance mechanisms

#### 3.2.1 Amyloid cascade hypothesis

The most widely accepted AD hypothesis is the amyloid cascade hypothesis, which was first proposed by Hardy and Allsop in 1991. This theory posits that Aβ plaques are initially deposited in the hippocampus and basal forebrain. They then accumulate additional Aβ peptides, forming insoluble aggregates. These aggregates induce mitochondrial damage, disrupt cellular homeostasis and cause synaptic dysfunction. This process establishes Aβ deposition as the pivotal trigger of AD pathogenesis. Aβ exists in two primary forms: monomeric/oligomeric species with low molecular weight that can diffuse through neural structures such as synapses (regarded as the main toxic form) and fibrillar aggregates formed through β-sheet folding, which ultimately assemble into plaques. Under physiological conditions, Aβ production and clearance maintain a dynamic equilibrium. However, pathological alterations in either production or clearance rates disrupt this balance, resulting in excessive cerebral Aβ accumulation. This accumulation triggers pathological cascades, including oxidative stress, NFT formation, and mitochondrial dysfunction. Critically, these processes further amplify Aβ accumulation, thereby creating a self-propagating cycle (Hardy and Allsop 1991).

#### 3.2.2 Nanomedicine treatment strategies

Inhibition of Aβ aggregation and oligomerization aims to prevent the initial formation or growth of toxic Aβ species. Chitosan/hyaluronic acid-glutaraldehyde coassembled nanoparticles (CHG NPs) exemplify this strategy by employing multifor force surface interactions (Wang et al. 2021). The abundant electron-rich functional groups on CHG NPs engage with Aβ oligomers via ionic bonds, hydrogen bonds, and n–π* interactions. These interactions induce conformational rigidification, slow fibril elongation, and alter the aggregation pathway. *In vivo* validation in *Caenorhabditis elegans* CL2006 models demonstrated the potential of CHG NPs in inhibiting Aβ deposition.

A complementary inhibitory strategy focused on the earliest nucleation events. KLVFFAED peptide-modified iron-rhein/polydopamine nanoparticles (K8@Fe-Rh/PDA NPs) utilize a polydopamine-based surface to interact with Aβ monomers through catechol and π–system interactions, effectively inhibiting the aggregation of Aβ oligomers (Yin et al. 2023). Beyond direct antiaggregation, this system was also reported to activate the sirtuin 1/peroxisome proliferator-activated receptor gamma coactivator 1-alpha (SIRT1/PGC-1α) pathway, promoting mitochondrial biogenesis and suppressing neuronal apoptosis in cellular models, suggesting additional neuroprotective benefits.

Targeting existing fibrils for disassembly is a key strategy to reverse established pathology. For example, Wei et al. (2024) developed QUE and p-phenylenediamine (p-PD)-derived red-emitting CDs (R-CDs) for multitarget AD therapy. Specifically, aromatic moieties, phenolic hydroxyl groups, and amino functionalities on R-CDs mediate robust interactions with Aβ, achieving rapid depolymerization of mature fibrils (<4 h) at micromolar concentrations (2–5 μg·mL^−1^) and scavenging excess ROS. Consistent with previous reviews (Wang et al. 2024), the cooperative action of π–π stacking and hydrogen bonding is a key mechanism enabling both fibril depolymerization and ROS clearance.

AuNP- and PEG-modified metal‒organic frameworks (AuNPs@PEG/MIL-101) have also been shown to disrupt preformed fibrils and inhibit new fibrillation *in vitro* (Yang et al. 2023). This proof-of-concept study demonstrated that such modulation of the monomer‒fibril equilibrium could mitigate Aβ-induced cytotoxicity in PC12 cells, highlighting a potential route to reduce the fibrillar burden.

Similarly, RVG29-functionalized biodegradable mesoporous silica nanoparticles loaded with ultrasmall cerium oxide nanocrystals (RVG29-bMSNs@Ce-1F12) promoted the gradual transformation of β-sheets and typical fibrillar structures into short linear β-sheets and irregular spherical structures (Zhang et al. 2024). Over time, the short linear β-sheets continuously decreases, indicating that it can facilitate the depolymerization of insoluble Aβ_42_ fibrils and shift the system toward a dynamic equilibrium between Aβ monomers and protofibrils, while also reducing Aβ_42_ aggregation-induced ROS production and microglial activation.

Nanoplatforms can also reduce the Aβ burden by increasing its clearance through indirect biological pathways. Dual-targeting layered double hydroxide nanoparticles (LRsAR) operate by silencing genes critical to Aβ production, namely, *BACE1* or *GSK3β* (Zhang et al. 2023). This gene-silencing approach resulted in reduced Aβ plaque burden and ROS levels while promoting plaque degradation in model systems.

**Figure 6.**
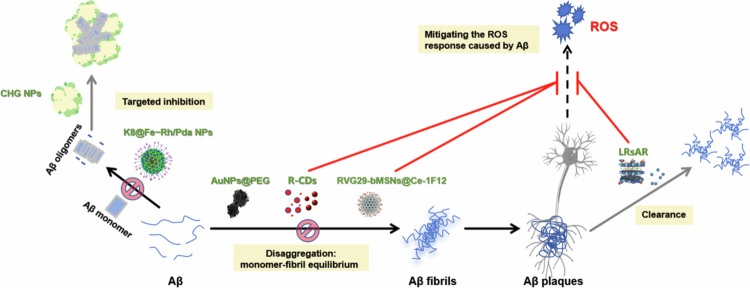
Nanomedicine strategies targeting Aβ pathology in AD. Schematic overview of engineered nanoplatforms that intervene in Aβ aggregation and toxicity through: inhibition of Aβ oligomerization and fibril formation (CHG NPs (Wang et al. 2021), K8@Fe-Rh/PDA NPs (Yin et al. 2023); disaggregation of pre-formed Aβ fibrils and plaques via modulation of monomer-fibril equilibrium (AuNPs@PEG (Yang et al. 2023), R-CDs (Wei et al. 2024), RVG29-bMSNs@Ce-1F12 (Zhang et al. 2024); clearance by gene silencing of Aβ-producing enzymes (LRsAR (Zhang et al. 2023); and scavenging of Aβ-induced ROS. Created with Bioicons.com and SciDraw.io.

In summary, grounded in the Aβ cascade hypothesis, nanomedicine strategies employ engineered platforms to disrupt Aβ pathogenesis through targeted inhibition, disaggregation, and clearance mechanisms while also mitigating Aβ-induced ROS responses, as depicted in Figure 6.

### 3.3. Targeted nano-delivery systems modulating tau phosphorylation, inhibiting fibrillization, and preserving neuronal integrity

#### 3.3.1. Tau protein hypothesis

Another mainstream hypothesis for AD pathogenesis is the tau hypothesis. In 1988, Wischik et al. isolated tau protein from AD patient brain tissue and subsequently identified it as a principal constituent of paired helical filaments within NFTs, suggesting its potential causative role in AD (Wischik et al. 1988). Subsequent research established that NFTs, a core pathological hallmark of AD, arise from the abnormal aggregation of neuronal cytoskeletal elements, primarily hyperphosphorylated tau protein. Under normal physiological conditions, the tau protein binds to tubulin and promotes microtubule assembly and stability. This binding is crucial for maintaining neuronal structure and facilitating axonal transport. However, the hyperphosphorylation of tau critically impairs its affinity for microtubules, leading to detachment. Consequently, this dissociation results in microtubule destabilization. This instability, in turn, induces axonal transport impairment and ultimately contributes to nerve fiber degeneration, which is the key process implicated in AD pathogenesis (Chen and Yu 2023).

#### 3.3.2. Nanomedicine treatment strategies

**Figure 7.**
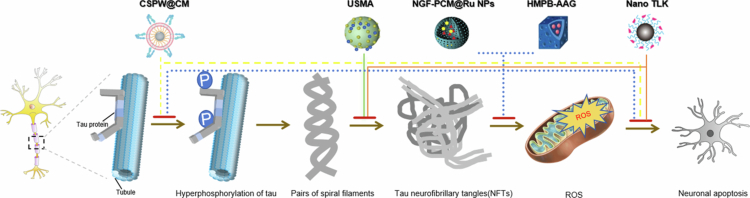
Nanomedicine strategies targeting tau pathology in AD. Schematic overview of engineered nanoplatforms that intervene in tau hyperphosphorylation, aggregation, and neurotoxicity: CSPW@CM NPs (Xu et al. 2025) promotes the degradation of hyperphosphorylated tau and ameliorates cognitive dysfunction; USMA (Qiao et al. 2023) suppresses tau NFT aggregation; NGF-PCM@Ru NPs (Yuan et al. 2023) and HMPB-AAG (Tang et al. 2022) reduce tau hyperphosphorylation, oxidative stress and inhibit apoptosis; Nano-TLK (Zhu et al. 2021) suppresses the formation of NFTs and alleviates tau-mediated apoptosis. These strategies collectively disrupt the tau pathological cascade while mitigating ROS-mediated neuronal apoptosis. Created with Bioicons.com and SciDraw.io.

Consequently, in response to this pathogenic mechanism, researchers have developed diverse nanomedicine strategies for AD treatment, as shown in Figure 7. To modulate tau pathology, nanomedicine strategies have been developed to inhibit aggregation, promote clearance, and provide neuroprotection in a series of preclinical studies.

To this end, Xu et al. (2025) reported that wogonoside-functionalized Cu_2−*x*_Se nanoparticles (CSPW@CM NPs) can activate the ubiquitin-proteasome pathway to inhibit ubiquitin-specific protease 14 in neurons, which promotes the degradation of hyperphosphorylated tau and ameliorates cognitive dysfunction in AD mice. Complementing this, Qiao et al. (2023) developed a USMA that releases methylene blue, which inhibits the aggregation of the short peptide VQIVYK (a core fragment critical for tau aggregate formation). Simultaneously, the charge interaction between the AuNPs and tau protein contributes to the suppression of tau NFT aggregation. Beyond small molecules, protein-based strategies such as NGF delivery via NGF-PCM@Ru NPs have shown efficacy in inhibiting tau hyperphosphorylation, reducing oxidative stress, and improving cognitive function in AD models (Yuan et al. 2023).

In expanding the therapeutic scope, Tang et al. (2022) engineered hollow mesoporous Prussian blue nanoparticles (HMPB NPs) loaded with the high-affinity Hsp90 inhibitor 17-AAG (HMPB-AAG nanoagents) for the combined treatment of tauopathy-induced AD. Their results demonstrated that HMPB-AAG nanomedicine inhibits Hsp90 through 17-AAG. It also facilitates the recognition of p-tau by the Hsp40/Hsp70 complex and promotes the subsequent degradation of p-tau via the CHIP-mediated ubiquitin-proteasome system. This treatment led to a reduction in ROS generation, downregulation of lipid peroxidation markers, 4-hydroxynonenal (4‑HNE), phosphorylated extracellular signal-regulated kinase, and key proapoptotic proteins—i.e. Bcl-2-associated X protein and cysteine aspartate-specific protease 3. Consequently, HMPB NPs reduced the accumulation of p-tau. and inhibited apoptotic cell death. Simultaneously, it decreased the release of proinflammatory factors (interleukin‑1β [IL‑1β] and TNF‑α) and mitigated neuroinflammation and the progression of p‑tau pathology.

Additionally, Zhu et al. (2021) designed a targeted multifunctional nanoinhibitor based on self-assembled polymer micelles modified with a tau-binding peptide. The tau-targeted (D)-TLKIVW peptide (TLK)-based nano-TLK stably binds to the tau protein through multivalent interactions at multiple surface sites. This binding inhibits the conformational transition of the tau protein into β-sheet structures and effectively suppresses NFT formation. Nano-TLK also recognizes tau aggregates and prevents their seeding in neuronal cells, significantly alleviating tau-mediated apoptosis.

### 3.4 Engineered nanocarriers for targeted AChE inhibition and synergistic cholinergic modulation

#### 3.4.1 Cholinergic injury hypothesis

In 1971, J. Anthony Deutsch clearly proposed an important relationship between cholinergic function and synapses and memory sites (Deutsch 1971). Later, in 1982, Raymond T. Bartus and Reginald L. Dean elucidated that significant cholinergic dysfunction occurs in the CNS during old age and dementia. They demonstrated a relationship between these changes and memory loss, noting that similar memory deficits can be artificially induced by blocking cholinergic mechanisms in young subjects. Conversely, under tightly controlled conditions, cholinergic stimulation reliably improves memory in aged subjects, further supporting the cholinergic impairment hypothesis (Bartus et al. 1982). The cholinergic injury theory is now widely accepted. It holds that cholinergic neurons in the basal forebrain synthesize large amounts of ACh. These neurons are primarily located in the basal nucleus of Meynert, the diagonal band nucleus, and the medial septal nucleus. The ACh is transported to the cerebral cortex and hippocampus via projection fibers. It is believed to be involved in learning and memory, and the hippocampus serves as a crucial anatomical substrate for these processes (Wu et al. 2021).

#### 3.4.2 Nanomedicine treatment strategies

Inhibiting AChE activity prevents ACh hydrolysis, thereby preserving its physiological function. Clinically used AChE inhibitors such as neostigmine and donepezil exemplify this approach. Nanomedicine enhances the therapeutic potential of AChE inhibition through various strategies.

For example, dcHGT NPs, which are coloaded with donepezil and chloroquine in albumin nanoparticles, led to decreased markers of ACh‑related neuroglial inflammation and Aβ‑induced inflammatory factors (TNF‑α and interferon‑γ). Additionally, the administration of these NPs was linked to increased expression of synaptic remodeling proteins (synaptophysin, growth-associated protein 43), improved neuronal morphology, and mitigated memory deficits, pointing to a potential enhancement of ACh regulation *in vivo* (Georgieva et al. 2023). In a different approach, Naila et al. (Ouyang et al. 2022) reported that Ag/Au BNPs function as nanozymes, exhibiting competitive AChE inhibition (increasing K_m_ by 288–1185.7%) through structural mimicry and hydrophobic interactions.

To rapidly assess *in vivo* efficacy and biodistribution, Oliveira et al. engineered an extracellular vesicle (EV) nanosystem to improve the targeting and efficacy of AChE inhibitors. Their investigation demonstrated that AChE inhibitors encapsulated within EVs are efficiently transported into cells. The primary transport pathways are macropinocytosis and clathrin-dependent endocytosis. Consequently, EV-mediated delivery of donepezil suppresses AChE enzyme activity in the heads of zebrafish larvae more pronouncedly than the free drug (Oliveira Silva et al. 2024).

### 3.5 Mitigating ROS-mediated oxidative stress and enhancing neuroprotection

#### 3.5.1 Oxidative stress hypothesis

The oxidative stress hypothesis posits that AD is driven by a pathological imbalance in which overproduction of ROS overwhelms endogenous antioxidant defenses. The brain is especially vulnerable to such damage due to its high lipid content, elevated oxygen demand, and relatively limited antioxidant capacity. With aging, oxidative damage progressively accumulates. ROS readily attack key biomolecules, leading to lipid peroxidation, protein carbonylation, nucleic acid oxidation, and the formation of advanced glycation end products. These modifications contribute to neuronal dysfunction and ultimately cell death. In AD, specific sources of ROS have been identified, including mitochondrial dysfunction, altered superoxide dismutase activity, monoamine oxidase metabolism, and disrupted iron homeostasis. Strong evidence supports the presence of oxidative stress in AD brains, reflected in elevated biomarkers such as 4-HNE, malondialdehyde (lipid peroxidation), and 8-hydroxy-2ʹ-deoxyguanosine/8-hydroxyguanosine (nucleic acid damage) (Benzi 1995). While it remains debated whether oxidative stress is a primary trigger or a secondary consequence of AD pathology (Markesbery 1997), ROS are consistently implicated in neurodegeneration. Therefore, therapeutic strategies aimed at reducing oxidative damage offer a promising avenue for ameliorating the AD brain microenvironment.

#### 3.5.2 Nanomedicine treatment strategies

For example, Wang et al. (2023) designed a lipoprotein-like nanocomposite (RLA-rHDL@ANG) comprising recombinant high-density lipoprotein (rHDL) and an apolipoprotein-derived peptide (RLA) with Aβ-binding and scavenging capabilities. The analysis results indicated that RLA-rHDL@ANG possesses both ROS sensitivity and scavenging properties. This nanocomposite slows rapid caspase-dependent neuronal apoptosis, which is triggered by H₂O₂-induced oxidative reactions.

Similarly, Guo et al. (2021) synthesized selenium quantum dots (SeQDs) as a nanosystem with robust free radical scavenging activity. This system confers protection against diverse oxidative stressors *in vitro* and displays broad-spectrum antioxidant efficacy. Notably, the SeQDs effectively attenuate tau protein hyperphosphorylation in AD models. They achieve this by downregulating phosphorylation at the PHF1 (Ser396/404) and CP13 (Ser202) antibody epitopes. This effect further diminishes oxidative stress and restores neuronal function.

In parallel, Dou et al. (2021) leveraged plant-derived therapeutics to combat oxidative stress by encapsulating QUE within human serum albumin (HSA), creating HSA@QUE nanoparticles (HQ NPs) as potential natural antioxidant nanotherapies for treating advanced AD. These HQ NPs exhibit excellent radical-scavenging activity. They effectively reduce 4-HNE levels and downregulate the expression of apoptosis-related proteins (e.g. the tumor protein p53 and caspase-3). These findings demonstrate their strong antioxidant capacity and ability to improve cognitive function in an AD model.

### 3.6 Multiplexed nanotherapeutic targeting of mitochondrial cascade dysfunction

#### 3.6.1 Mitochondrial dysfunction hypothesis

Research indicates that mitochondrial structure and function are abnormal in the brains of AD patients. As mitochondria provide the primary cellular energy supply, abnormalities in bioenergetic enzymes or functions result in insufficient energy availability, particularly in the brain, thereby causing neuronal damage. Moreover, impaired mitochondrial axonal transport prevents efficient energy delivery from neuronal cell bodies to synapses, which leads to aberrant synaptic plasticity—a critical mechanism underlying learning and memory. Supporting this, studies have demonstrated that restoring mitochondrial function through phosphatidylglycerol supplementation ameliorates neuronal synaptic disorders (Kawatani et al. 2024).

Furthermore, mitochondria represent a primary site for disease initiation and propagation within cells. Metabolic perturbations may lower the threshold for irreversible damage from sublethal metabolic stressors. Consequently, diverse mitochondrial abnormalities ultimately contribute to oxidative stress, bioenergetic failure, and disrupted cytosolic calcium homeostasis. These pathological changes establish a self-amplifying cycle of mitochondrial dysfunction that drives neurodegenerative processes in AD (Gibson et al. 2010).

#### 3.6.2 Nanomedicine treatment strategies

**Figure 8.**
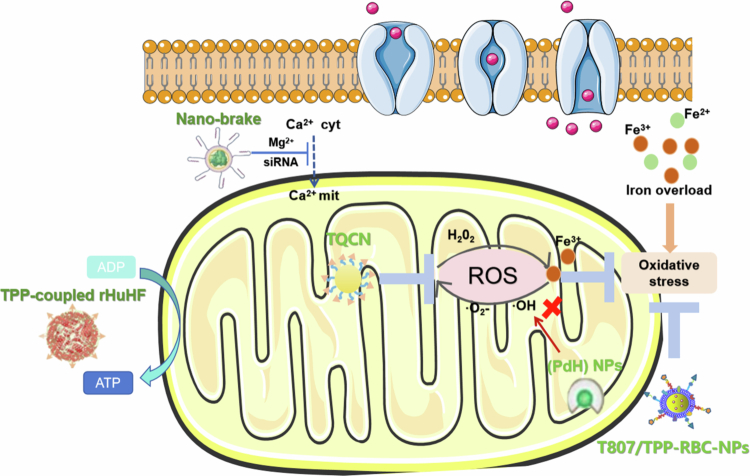
Nanomedicine strategies targeting mitochondrial dysfunction in AD. Schematic overview of nanoplatforms addressing key pathological mechanisms: nanobrake (Zhang et al. 2023)—Mg^2+^ and *CypD*-siRNA codelivery prevents mitochondrial Ca^2+^ overload and mPTP opening. TPP-rHuHF (Xia et al. 2024)—lycopene-loaded nanocages enhance ATP production and preserve mitochondrial DNA. TQCN (Thangwaritorn et al. 2024)—mitochondria-targeted QUE chelates Fe^3+^, suppressing Fenton reaction-induced ROS. PdH NPs (Zhang et al. 2019)—sustained H₂ release scavenges ·OH, restoring redox homeostasis. T807/TPP-RBC-NPs (Gao et al. 2020)—CUR-loaded carriers with dual targeting, attenuate oxidative damage and apoptosis. These strategies collectively disrupt bioenergetic failure, calcium dysregulation, and oxidative stress in AD-related mitochondrial dysfunction. Created with Bioicons.com and SciDraw.io.

Oxidative stress-induced mitochondrial dysfunction is a key contributor to AD pathogenesis, making it a promising target for nanomedicine. Researchers have therefore developed multiple strategies to intervene in this process (Figure 8).

To directly enhance mitochondrial bioenergetics and integrity, TPP-coupled rHuHF was engineered for neuronal mitochondrial targeting. In an aging model, this intervention was shown to sustain ATP production, preserve mitochondrial DNA and ultrastructure, and inhibit pathological mPTP opening, which correlated with the rescue of synaptic deficits and improved memory (Xia et al. 2024).

Notably, mitochondrial calcium ion (Ca^2+^) dysregulation and mPTP dysfunction are key upstream events in the mitochondrial cascade of AD. To address this issue, Zhang et al. (2023) proposed a ‘double-hit brake’ nanotherapeutic strategy. This strategy combines two components: magnesium ions (Mg^2+^), natural antagonists of Ca^2+^, and siRNA, which silences the gene for cyclophilin D (*CypD*)—a key regulator of the mPTP. This approach synergistically prevented mitochondrial Ca^2+^ overload and inhibited mPTP opening, thereby halting the mitochondrial dysfunction cascade. Consequently, this nanobrake therapy effectively attenuated dysfunction in cerebrovascular endothelial cells, neurons, and microglia, alleviating AD neuropathology and rescuing cognitive impairment.

Furthermore, redox-active iron ions (Fe^3+^) accumulate in mitochondria, triggering Fenton reactions, impairing electron transport chain activity, and inducing excessive ROS production. ROS further attack polyunsaturated fatty acids in neuronal membranes, leading to lipid peroxidation and structural damage. Liu et al., (2024) constructed a mitochondria-targeted QUE smart nanosystem (TQCN). This nanoplatform was preferentially targeted to and enriched in mitochondria, chelated labile iron ions, inhibited iron-mediated Fenton reactions, alleviated oxidative stress, and restored mitochondrial homeostasis. These results highlight the key role of the nanosystem in regulating the mitochondrial iron balance and oxidative stress.

Additionally, Zhang et al. (2019) utilized molecular hydrogen (H_2_) as a selective antioxidant to scavenge highly cytotoxic ROS such as hydroxyl radicals (·OH), demonstrating the potential for mitigating oxidative stress in AD treatment. However, to overcome the challenges of low hydrogen solubility and poor sustained accumulation in the brain, they developed hydrogenated palladium nanoparticles (PdH NPs). These NPs enable high hydrogen loading capacity and provide sustained in situ hydrogen release within the AD brain. The released H_2_ exhibits potent reducing activity, facilitating efficient autocatalytic removal of cytotoxic ·OH. Critically, this reductive activity restores mitochondrial function and enhances neuronal energy metabolism by alleviating oxidative stress and activating antioxidant pathways. It ultimately ameliorates cognitive dysfunction in AD mouse models.

Building on antioxidant delivery strategies, Gao et al. (2020) designed a strategy to deliver the antioxidant CUR to neuronal mitochondria. To achieve this, they encapsulated CUR in human serum albumin NPs camouflaged with RBC membranes. Subsequent surface modification with T807 (for neuronal targeting) and triphenylphosphonium (TPP; for mitochondrial targeting) yielded the functionalized nanocapsule T807/TPP-RBC-NPs. Their data confirmed that CUR-loaded nanocarriers alleviate AD pathology by reducing mitochondrial oxidative stress and inhibiting neuronal apoptosis.

### 3.7 Targeted disruption of pathological copper nexus via dual-modal chelation and photothermal ablation

#### 3.7.1 Imbalance in metal ion metabolism hypothesis

Metal ions, particularly zinc, iron, and copper, significantly contribute to neurodegeneration by disrupting protein folding and promoting oxidative stress. Critically, disrupted metal homeostasis represents a well-established pathological mechanism in neurodegenerative diseases. Zinc ions (Zn^2+^) modulate Aβ self-assembly kinetics and amyloid morphology via specific coordination sites. Zn^2+^ promotes Aβ aggregation through both intramolecular and intermolecular binding. Notably, the accumulation of synaptic Zn^2+^ in excitatory synapses can induce Aβ oligomer formation (Kozlowski et al. 2012).

Similarly, redox-active iron ions (Fe^2+^/Fe^3+^) catalyze Fenton reactions that generate oxidative stress, accelerating AD pathogenesis (Mo et al. 2025). Copper ions (Cu^2+^) exhibit analogous redox activity, producing ROS that trigger cellular stress responses. Specifically, copper demonstrates high affinity for APP, forming stable Cu^2+^–Aβ complexes. Overload of these complexes generates neurotoxic compounds that induce oxidative stress, neuronal degeneration, and cognitive dysfunction. Although Cu^2+^ is physiologically essential for neurotransmitter synthesis and mitochondrial respiration, copper imbalance is implicated in AD and related neurodegenerative disorders (Zhang et al. 2023).

#### 3.7.2 Nanomedicine treatment strategies

For instance, Chen et al. (2023) developed polydopamine nanoparticles (PDA NPs) that effectively inhibit copper-mediated Aβ aggregation, reduce Aβ–Cu^2+^ complex formation, and scavenge ROS generated by copper or Aβ–Cu^2+^ complexes. This mechanism promotes cell survival by lowering intracellular ROS levels. Notably, the PDA NPs depolymerize the preformed Aβ‒Cu^2+^ complexes. Furthermore, their high photothermal conversion efficiency enables NIR light irradiation to degrade these complexes, substantially reducing Aβ plaque deposition in copper-rich environments and extending the lifespan in an AD nematode model.

Beyond this multifunctional platform, researchers have mitigated copper-associated Aβ toxicity using potent chelating agents to protect neuronal cells. For instance, Du et al. (2022) engineered a two-dimensional niobium carbide nitride nanochelator that inhibits copper-induced Aβ peptide aggregation and scavenges excess cellular ROS through intrinsic antioxidant activity. In a similar vein, Liu et al. (2024) demonstrated that carbon quantum dots (CQDs) function as effective copper chelators. These CQDs not only prevent copper-mediated Aβ aggregation but also dissolve existing Aβ fibrils via NIR laser-induced photothermal effects.

Concurrently, Li et al. (2023) reported a nanocomposite that acts as an efficient and highly selective copper chelator. This material regulates the enzymatic activity of both free copper ions and Aβ–Cu^2+^ complexes, reduces pathological oxidative stress, and exerts anti-inflammatory effects. Critically, it also significantly inhibits copper-mediated Aβ aggregation and protects neurons from the toxicity of Aβ‒Cu^2+^ complexes in transgenic *Caenorhabditis elegans* CL2120.

### 3.8 Precision remodeling of gut-brain axis crosstalk via neuroimmune-metabolic modulation

#### 3.8.1 Neuroinflammation hypothesis and microbiota hypothesis

The neuroinflammation hypothesis posits that chronic activation of the brain's immune response is a central driver of AD progression. Early evidence, such as the presence of activated microglia in postmortem AD brains, suggested immune involvement. Recent research solidifies this link, showing that neuroinflammation—primarily mediated by activated microglia and astrocytes—propagates a vicious cycle. This cycle involves Aβ deposition, tau pathology, proinflammatory cytokine release, and subsequent neuronal damage, particularly in early disease stages (Calsolaro and Edison 2016; Jia et al. 2020). Neuroinflammation is now recognized as both a potential diagnostic biomarker and a promising therapeutic target.

The gut microbiota hypothesis is intrinsically connected. Dysbiosis, or an imbalance in the gut microbial composition, can increase intestinal permeability, leading to systemic immune activation and chronic peripheral inflammation. This inflammation can compromise the BBB and promote neuroinflammation through various pathways, potentially accelerating neurodegeneration. Furthermore, molecular mimicry between bacterial and host amyloids might promote protein misfolding and microglial activation via the gut‒brain axis (Kowalski and Mulak 2019). Studies have confirmed a bidirectional communication pathway along this axis, where a proinflammatory gut state can exacerbate neuroinflammation and impair neuronal function (Bairamian et al. 2022).

Collectively, these interconnected hypotheses suggest that AD pathology is fueled by a self-reinforcing loop of central neuroinflammation and peripheral inflammatory signals originating from the gut. Therefore, therapeutic strategies that simultaneously target neuroinflammation and modulate the gut microbiota may offer a synergistic approach to mitigate AD progression.

#### 3.8.2 Nanomedicine treatment strategies

Microbial metabolites such as short-chain fatty acids, trimethylamine N-oxide, and gasotransmitters influence BBB permeability and modulate neuroimmune responses (Mustafa et al. 2025). Furthermore, recent cross-omics integration data from CSF indicate that circulating triacylglycerols are involved in regulating immune cell activity and inflammation, while alterations in glycosphingolipids may reflect demyelination and remyelination mediated by proteasomal degradation of secreted phosphoprotein 1 in the CSF. The degradation of sphingolipids leads to an increase in very long-chain (C22–C26) galactosylceramide and sulfatide, which further promotes active remyelination of neuronal connections (Jiang et al. 2025). With respect to neuroinflammation regulation, studies have shown that activation of the brain-derived neurotrophic factor-tropomyosin receptor kinase B signaling pathway can suppress the activation of microglia (ionized calcium-binding adapter molecule 1) and astrocytes (glial fibrillary acidic protein), reduce the expression of inflammatory factors, and thereby attenuate inflammation, decrease neuronal damage, and upregulate cognitive function (Luo et al. 2019).

Therefore, to investigate gut‒brain axis modulation in AD, Yang et al. (2022) synthesized a TGNYKALHPHNG peptide-functionalized nanodrug (TG-CS/DMY@SeNPs). This targeted compound crosses the BBB and inhibits Aβ aggregation more effectively than the nontargeted control (CS/DMY@SeNPs). It also suppresses inflammatory cytokine secretion via the NF-κB signaling pathway in *APP/PS1* mouse brains. Additionally, TG-CS/DMY@SeNPs restored intestinal barrier integrity and modulated the inflammation-associated gut microbiota (*Bifidobacterium*, *Dubosiella*, and *Desulfovibrio*). Notably, only TG-CS/DMY@SeNPs increased the relative abundance of *Corynebacterium gordonii*, subsequently downregulating the expression of the NOD-like receptor family pyrin domain-containing protein 3 (NLRP3) inflammasome and reducing serum inflammatory factor levels. Collectively, these findings demonstrate the amelioration of neuroinflammation via the gut microbiota-inflammasome-brain axis.

Building on this, Guo et al. (2023) demonstrated that orally administered chiral Au NPs interact with the gut microbiota via electrostatic interactions. This interaction increases tryptophan binding and enhances its biotransformation. The NPs also upregulate genes encoding tryptophan metabolism-related enzymes (e.g. IaaM, IaaH, amino acid oxidase, and IaaDH), thereby elevating the level of IAA. Concurrently, IAA activates the aryl hydrocarbon receptor in brain microglia and astrocytes and suppresses the NLRP3 inflammasome via the transcription factor *NF‑κB*, thereby alleviating pathological symptoms in AD mice.

Further expanding the scope, Qu et al. (2021) reported that honokiol nanomedicine (Nano-HO) protected against cognitive deficits in TgCRND8 mice. Nano‑HO inhibited neuroinflammatory responses (TNF-α, IL‑1β) and suppressed the activation of microglia, astrocytes, and Aβ plaque burden. Simultaneously, it modulates the composition and structure of the gut microbiota, restoring bacterial populations such as *Faecalibacterium* and *Actinobacteria*, thereby protecting the integrity and stability of the gut microbiome. Nano-HO mitigated AD pathology by regulating APP processing, preventing tau hyperphosphorylation, and modulating the JNK/CDK5/GSK‑3β signaling pathway.

### 3.9 Reconstituting estrogenic neuroprotective networks via ERβ-mediated signalosome activation

#### 3.9.1 Estrogen deficiency hypothesis

The estrogen deficiency hypothesis posits that the decline in estrogen levels, particularly after menopause, contributes significantly to the pathogenesis of AD. Estrogen exerts multifaceted neuroprotective effects: it promotes neuronal repair and function, enhances the synthesis of key neurotransmitters (e.g. ACh, dopamine, and serotonin) and the uptake of glutamate, dampens inflammation, improves cerebral blood flow, facilitates Aβ clearance, supports oligodendrocyte viability, and modulates various neuroprotective pathways (Preciados et al. 2016; Alhajeri et al. 2026).

At a molecular level, estrogen upregulates seladin-1, a protein associated with neuronal resistance. This upregulation stimulates cellular cholesterol synthesis, rendering neurons more resistant to oxidative stress and Aβ toxicity, suggesting that seladin-1 as a potential mediator of estrogen benefits (Peri 2016). Furthermore, estrogen regulates immune modulation within the brain by reducing the S-nitrosylation (SNO) of complement component 3 (C3). The decline in estrogen after menopause permits excessive C3 SNO, which in turn enhances microglial-mediated synaptic pruning, leading to synaptic loss and cognitive decline (Yang et al. 2022). This hypothesis underscores the potential of estrogen-based or estrogen-mimetic therapeutic strategies, particularly in addressing the heightened AD risk observed in postmenopausal women.

#### 3.9.2 Nanomedicine treatment strategies

According to prior reviews, factors such as gender-dependent immunoreactivity, autonomic nerve regulation, and the perimenopausal decline in estrogen collectively heighten the susceptibility of the locus coeruleus to inflammatory disruption in women. This heightened susceptibility underscores the importance of understanding the interplay between sex biology and neural circuits to identify potential windows for early therapeutic intervention in AD (Stapleton et al. 2026).

Based on preclinical evidence, Chen et al. (2024) demonstrated that a multifunctional phytoestrogen nanoregulator (LQ-LP) shows promise for the treatment of AD in estrogen-deficient contexts, such as in postmenopausal females. This nanomedicine inhibits the progression of estrogen-deprived AD through two primary pharmacological mechanisms: First, it enhances synaptic protection and exerts antiapoptotic effects by activating the estrogen receptor β/brain-derived neurotrophic factor/extracellular signal-regulated kinase signaling pathway. Second, it suppresses pathological processes induced by glutamate excitotoxicity, specifically attenuating Ca^2+^ influx peaks, reducing oxidative damage, and ameliorating cholinergic dysfunction. Collectively, these coordinated actions achieve significant therapeutic outcomes against AD pathology, highlighting the potential of ERβ-targeted nanotherapies as a viable strategy for addressing estrogen deficiency-associated AD, particularly in postmenopausal women.

### 3.10 Translational prospects

The preceding sections detail a rich landscape of nanomedicine strategies for AD, predominantly validated in cellular and animal models. Table 3 summarizes selected approaches that have shown promising results in preclinical studies and may be closer to clinical translation, along with key challenges that must be addressed. It is crucial to interpret animal study results with caution, as efficacy in zebrafish and genetically engineered mouse models does not guarantee success in the more complex and heterogeneous human diseases. The path to the clinic requires rigorous assessment of pharmacokinetics, biodistribution, long-term safety, immunogenicity, and large-scale GMP production for these sophisticated nanoformulations.

**Table 3.** Nanomedicine approaches for AD with preliminary translational potential (preclinical stage).

Target pathogenesis	Nanocarrier system	Key design features for translational potential	Stage of development (AD animal models)- admin route	Major translational advantage	References
BACE1 silencing	Gal-NP@siRNA	Sequence-specific BACE1 siRNA delivery; GLUT1-mediated BBB transport	Late-stage preclinical (*APP/PS1* mice)-IV	Formulability; stability; and brain delivery capability	Zhou et al. (2020)
Aβ aggregation	RVG29-bMSNs@Ce-1F12	1F12 antibody-mediated specific binding to all Aβ_42_ species; RVG29 peptide targeting; Biodegradable mesoporous silica	Late-stage preclinical (*APP/PS1* mice)-IV	Fow toxicity and high biocompatibility;biodegradable;multi-functional	Zhang et al. (2024)
Tau hyperphosphorylation	CSPW@CM NPs	CSPW as USP14 nanoinhibitor; Activates ubiquitin-proteasome pathway	Early-stage preclinical (OKA-induced C57BL/6J mice)-IV.	Improved bioavailability; targeted delivery; and enhanced therapeutic effects with reduced dosage	Xu et al. (2025)
Cholinergic dysfunction	EVs-DNZ	Encapsulates donepezil for sustained release; Negative surface charge (~−42 to −43 mV) favoring BBB penetration	Early-stage preclinical (zebrafish larvae)-IV	Higher brain AChE inhibition efficacy; lower peripheral cholinergic side effects; and no toxicity	Oliveira Silva et al. (2024)
ROS-mediated oxidative stress	RLA-rHDL@ANG	ANG peptide-mediated LRP1-dependent BBB penetration; ROS-sensitive TK fragments for ROS scavenging	Early-stage preclinical (Aβ₄₂ hippocampal-injected AD mice)-IV	High brain accumulation (64.2 ng·g^−1^); lysosomal degradation promotion	Wang et al. (2023)
Mitochondrial dysfunction	Nano-brake	MMP9-activatable MAP peptide for targeted BBB penetration; co-encapsulates for ‘two-hit braking’	Late-stage preclinical (5xFAD mice)-IV	Synergistically halts mitochondrial dysfunction cascade; ameliorates neurovascular damage; and high biocompatibility (no organ toxicity)	Zhang et al. (2023)
Gut-brain axis/neuroinflammation	Tg-CS/DMY@SeNPs	Tg peptide-mediated BBB penetration; DMY modification with Se core	Late-stage preclinical (*APP/PS1* mice)-oral gavage	Regulates gut microbiota; reduces neuroinflammation; repairs gut barrier	Yang et al. (2022)
Estrogen deficiency	LQ-LPs	LQ encapsulation for ERβ-specific agonism; intranasal delivery for direct olfactory/trigeminal BBB bypass	Late-stage preclinical (ovariectomized *APP/PS1* mice)-intranasal	ERβ-selective neuroprotection; high brain bioavailability; no systemic estrogenic side effects	Chen et al. (2024)

Abbreviations: Gal-NP@siRNA: galactose-modified nanoparticles@small interfering RNA; RVG29-bMSNs@Ce-1F12: rabies virus glycoprotein 29-biodegradable mesoporous silica nanoparticles@cerium oxide nanocrystals-1F12 antibody; CSPW@CM NPs: wogonoside-functionalized ultrasmall Cu2-xSe nanoparticles; EVs-DNZ: vesicles–donepezil; RLA-rHDL@ANG: apolipoprotein E-derived RLA peptide-reconstituted high-density lipoprotein@angiopep-2; Nanobrake: MMP9-activatable cell-penetrating peptide-functionalized magnesium/cyclophilin D siRNA-loaded lipid nanocarrier; PDA NPs: polydopamine nanoparticles; Tg-CS/DMY@SeNPs: Tg peptide–chitosan–dihydromyricetin@selenium nanoparticles; LQ-LPs: liquiritigenin-loaded liposomes; and IV: intravenous.

## 4 The application of nanomedicine in the integrated diagnosis and treatment of AD

A translational medicine paradigm for nanomedicine development in AD involves a pathway from clinical pathological discoveries to the establishment of mechanistic hypotheses, followed by experimental platform validation and advancement toward clinical application. After analyzing the construction, delivery processes, multimodal mechanisms of action and translational potential of nanosystems, this section focuses on the integrated application value, current challenges, and future prospects of nanomedicine in the diagnosis and treatment integration for AD.

### 4.1 Clinical diagnosis

Clinical evidence indicates that Aβ protofibrils are key therapeutic targets in the pathogenesis and progression of AD. During the disease course, Aβ protofibrils represent the primary form exerting neurotoxicity, while mature amyloid plaques may exist in a relatively inert state (Johannesson et al. 2024). The significant efficacy of Donanemab (demonstrating a 35% slowing of disease progression) in patients with low tau protein burden further suggests that the clinical benefit window for plaque clearance exists in the early stages of the disease. By the time typical clinical symptoms manifest, substantial neuronal loss has often already occurred, and clearing tau pathology at this stage may be insufficient to reverse established structural damage (Vassar 2017). Therefore, the timing of the intervention is crucial. Early intervention during the preclinical phase or at the stage of MCI, or implementing preventive treatment for asymptomatic, Aβ-positive high-risk individuals, would hold significant clinical importance.

Owing to their outstanding physicochemical properties, NPs are ideal materials for constructing AD biomarker detection platforms. Nanoprobes targeting Aβ or tau proteins provide important tools for the early diagnosis of AD. As sensing elements, optical nanomaterials (e.g. Au NPs and quantum dots) enable highly sensitive detection of trace Aβ via surface plasmon resonance or fluorescence signal amplification. Electrochemical biosensing technology leverages the enhanced conductivity of carbon nanotubes, graphene, and other nanomaterials to amplify signals. This has facilitated the development of portable diagnostic devices and significantly improved the efficiency and accessibility of early AD screening. Furthermore, magnetic nanomaterials such as superparamagnetic iron oxide NPs can serve as contrast agents to specifically identify Aβ deposits in the brain via MRI, providing a basis for noninvasive diagnosis and visual monitoring of the disease (Sharma 2025). A summary of representative clinical studies is presented in Table 4.

**Table 4.** Representative clinical studies on the application of NPs in the early diagnosis of AD.

Types	Principle	Representative	Clinical application	Detection indicators	References
Optical sensing nanometers	Surface plasmon resonance or fluorescence signal amplification	Au/Ag NPs	Combined with 31-phosphorus magnetic resonance spectroscopy (^31^P-MRS) imaging to diagnose neurodegenerative diseases such as PD	The ratio of NAD^+^ to NADH was measured by ^31^P-MRS	ClinicalTrials.gov. NCT03815916 (2025)
		Quantum dot	Diagnostic kits	Fluorescence signals of Aβ and tau proteins in blood or tissue samples	Park et al. (2019)
Electrochemical biosensing nanomaterials	Enhanced conductivity effect	Carbon nanotubes	Composite platforms with high-density arranged carbon nanotubes	Blood samples of Aβ peptide and tau protein	Kim et al. (2020)
		Graphene	Graphene oxide electrochemical adapter	Human serum Aβ_42_	Vajedi et al. (2024)
Magnetic NPs	Magnetic field remote control	Superparamagnetic iron oxide nanoparticles (SPION)	ultrasmall SPION contrast agents (ferumoxytol injection)	MRI of the brain after intravenous injection	Azurity Pharmaceuticals (2025)

#### 4.1.1 Optical sensing nanomaterials

Common and widely used optical sensing nanomaterials include AuNPs/AgNPs and quantum dots (QDs). AuNPs/AgNPs offer good stability, simple synthesis, and flexible surface functionalization. These properties allow them not only to cross the BBB efficiently for targeted drug delivery but also to integrate multiple therapeutic mechanisms through surface modifications. Their unique optical properties, such as surface plasmon resonance, make them promising as highly sensitive contrast agents for diagnostics (Scarpa et al. 2023). For example, nanoplasmonic sensors can be built using antibody-modified AuNPs and guanidine hydrochloride. Here, guanidine hydrochloride disrupts protein hydrophobicity to expose antigenic epitopes. Binding to AuNPs then changes the surface plasmon resonance signal, enabling detection (Kim et al. 2019). Additionally, composite probes made from AuNPs and rose red dye combine surface-enhanced Raman scattering with fluorescence enhancement. These probes can quantify the Aβ concentration via Raman signal intensity and provide high-sensitivity fluorescence imaging of amyloid plaques in brain tissue. This offers a promising technical solution for early disease diagnosis (Xia et al. 2019).

Several clinical trials have explored AuNPs for diagnosing and treating neurodegenerative diseases. For example, a study on the effect of clean-surfaced nanocrystalline gold-8 (CNM-Au8) on neuronal redox states in PD patients has been completed (ClinicalTrials.gov. NCT03815916 2025). Similar approaches are being advanced for multiple sclerosis (ClinicalTrials.gov. NCT03993171 2025). However, research in this area faces challenges; some trials, such as one for ALS, have been withdrawn (ClinicalTrials.gov. NCT03843710 2025). Regarding safety, AuNPs may cause astrocyte proliferation, leading to neuroinflammation or excitotoxicity (Pereira et al. 2021). Other concerns include tissue accumulation causing hepatotoxicity (Zhang et al. 2015) and binding to DNA grooves, which could pose long-term risks such as genotoxicity, transcriptional interference, and epigenetic disruption (Niznik et al. 2024).

QDs have unique optical properties that overcome traditional dye and imaging limitations. These include tunable emission wavelengths, high fluorescence intensity, and photostability. They enable early AD detection by tracking Aβ aggregation *in vivo* or by accurately detecting biomarkers such as the *APOE* genotype (Chopra et al. 2022). Functionalization and coupling with specific biomolecules (e.g. aptamers, antibodies, peptides) allow QDs to specifically detect pathological biomarkers. Their intense, tunable, and persistent fluorescence *in vivo* helps detect biomarkers selectively at very low concentrations, supporting accurate early AD diagnosis (Sharma et al. 2025).

Researchers have developed diagnostic kits based on QDs. These kits use QDs as fluorescent NPs linked to oligonucleotides that recognize disease-related microRNAs or other markers, such as Aβ and tau proteins. When target molecules are present in a sample, the QDs enhance fluorescence, confirming the marker's presence. Studies have shown that this method is faster, more economical, and more effective than traditional techniques (Park et al. 2019). However, limitations remain. Heavy metal-based QDs may pose biocompatibility issues and proinflammatory effects (Sinha et al. 2024). Thus, further large-scale clinical application of QDs still requires rigorous trials to verify human safety and efficacy.

#### 4.1.2 Electrochemical biosensing nanomaterials

In electrochemical biosensing for AD diagnosis, the most representative nanomaterials are carbon-based, primarily carbon nanotubes (CNTs) and graphene. These materials significantly enhance biosensor performance due to their high electrical conductivity, large electroactive surface area, and versatile surface chemistry (Şak et al. 2025).

Carbon nanotubes are widely studied for applications such as biosensing and drug delivery owing to their excellent mechanical flexibility, quasi-ballistic electron transport, and tunable electronic properties (metallic or semiconducting) dependent on their chirality and diameter (Avouris et al. 2007). For instance, Kim et al. (2020) reported a composite sensing platform using densely aligned vertical CNTs, which detected Aβ peptides and tau proteins with high sensitivity. The platform demonstrated significant signal amplification, achieving detection limits much lower than those of bare electrodes. By measuring ratios such as t-tau/Aβ_42_, p-tau181/Aβ_42_, and Aβ_42_/Aβ_40_ in clinical blood samples, the sensor array successfully distinguished clinically diagnosed AD patients from healthy controls, with average sensitivity, selectivity, and accuracy approaching 90%. However, CNT-based sensors face several challenges, including limited long-term stability under varying storage conditions, insufficient validation of performance in large clinical cohorts, and poor reproducibility across manufacturing batches (Schneider et al. 2022).

Graphene and its derivatives, such as reduced graphene oxide (rGO), exhibit high mechanical strength, excellent electrical conductivity, and thermal stability, making them attractive for energy, wearable electronics, and sensing applications (Razaq et al. 2022). Vajedi and colleagues (Vajedi et al. 2024) developed an electrochemical aptasensor based on rGO for ultrasensitive detection of Aβ_42_ in human serum. The modified electrode showed an approximately sevenfold increase in the electroactive surface area and significantly accelerated the redox kinetics of Aβ_42_, yielding a peak current substantially higher than that of the bare electrode. However, the multistep fabrication process complicates production, increases costs, and limits scalability. Furthermore, inadequate assessment of the sensor's long-term stability and behavior in complex biological environments may impede clinical translation.

#### 4.1.3 Magnetic nanomaterials

Magnetic nanoparticles (MNPs) are promising for disease diagnosis and treatment due to their unique surface chemistry, good biocompatibility, inducible magnetic moments, and the possibility of remote magnetic manipulation for targeted delivery and separation (Rezaei et al. 2024).

Among various MNPs, superparamagnetic iron oxide nanoparticles (SPIONs) are particularly notable. Their biocompatibility, small size, relative safety, and unique superparamagnetic properties have led to their widespread use in preclinical models of amyloid-related diseases. Studies indicate that SPIONs with negatively charged or neutral coatings are better suited for *in vivo* imaging, as they minimize adverse effects such as protein corona formation while retaining effective magnetic performance (Mirsadeghi et al. 2016). Clinically, ultrasmall SPION contrast agents (e.g. ferumoxtran-10, ferumoxytol) have been administered intravenously to patients with CNS inflammatory diseases or lymphoma. Subsequent MRI scans provide enhanced visualization of lesions, aiding in surgical targeting (Farrell et al. 2013). Notably, Ferumoxytol (marketed as Ferabright) has received FDA approval as the first iron-based contrast agent for MRI to visualize brain lesions with impaired BBB in adults with known or suspected malignancies (Azurity Pharmaceuticals 2025). Nevertheless, concerns remain regarding potential tissue toxicity, inadequate renal clearance, and iron overload associated with SPIONs (Sanati et al. 2021; Lapusan et al. 2024). Ongoing research and clinical trials are needed to develop safer and more specific SPION formulations to fully realize their diagnostic and therapeutic potential.

### 4.2 Clinical treatment

Effective clinical management of AD requires early diagnosis with high sensitivity and low detection limits, while therapeutic success depends more on targeting precision than mere binding strength. For AD, excessively strong binding to Aβ does not necessarily improve clinical outcomes and may instead limit dosage escalation and increase safety risks due to complications such as amyloid-related imaging abnormalities (Boxer and Sperling 2023). Clinical evidence supports this: lecanemab, which selectively targets toxic Aβ protofibrils, demonstrates a more favorable efficacy-safety profile than broad-spectrum, high-affinity antibodies. In contrast, donanemab, while more potent, is associated with a higher incidence of ARIA, necessitating careful individual risk‒benefit assessment (van Dyck et al. 2023). These findings underscore the importance of optimizing both targeting specificity and clearance efficiency—a goal that nanomedicine strategies are designed to address. Nanosystems can cross the BBB in a controlled manner via self-assembly and biocompatible encapsulation, which may reduce vascular inflammation and BBB disruption associated with some monoclonal antibodies (Blockx et al. 2016). Additionally, their ability for stimulus-responsive and precise drug release minimizes off-target effects. When combined with early diagnostic data, these nanosystems enable coordinated multitarget therapy against Aβ, tau, and neuroinflammation, thus addressing the significant heterogeneity of AD.

Despite many sophisticated nanoplatforms in preclinical research, few nanomedicines have reached clinical trials or applications for AD. The following section will systematically review the clinical progress of nanomedicines based on strategies involving AChE inhibitors, siRNA/gene therapy, and antioxidants.

#### 4.2.1 Nanodelivery of AChE inhibitors

Currently, AChE inhibitors such as donepezil and rivastigmine remain first-line drugs for the clinical treatment of AD. Although these inhibitors have been on the market, these compounds are hindered by poor oral bioavailability, limited BBB penetration, and peripheral side effects, challenges (Hansen et al. 2008) that nanocarriers can overcome by enabling targeted brain delivery, controlled release, and reduced toxicity. While related patents exist—such as one for AChE inhibitor-coated NP conjugates (PCT/IN2015/050057) for treating neurodegenerative diseases—this strategy remains primarily in the preclinical research stage.

#### 4.2.2 Immunotherapies based on nanocarriers

As of 2025, three monoclonal antibodies targeting Aβ—aducanumab, lecanemab, and donanemab—have received FDA approval for early AD treatment, marking a major breakthrough in disease-modifying therapy. However, these antibodies face limitations, including restricted BBB permeability and a higher risk of inducing ARIA. Recent studies have employed a ‘Trojan horse’ strategy, utilizing transferrin receptors on cerebrovascular endothelial cells as natural conduits to help antibodies enter the brain parenchyma more precisely and safely (Pizzo et al. 2025). These findings suggest that NP-based delivery systems designed for specific and highly efficient BBB penetration could significantly improve the safety and efficacy of next-generation Aβ immunotherapies. Most related research, however, is still confined to animal models (Bukhary 2024). In terms of intellectual property, patents have been filed for nanoimmunocomplexes containing CpG oligodeoxynucleotides (CpG ODN) (CN119607004A), which modulate immune responses by activating Toll-like receptor 9, offering new avenues for treating neurological disorders such as AD.

#### 4.2.3 NP-based siRNA or gene therapies

Small-molecule drugs and immunotherapies primarily aim to alleviate symptoms or slow disease progression but rarely offer a cure. Consequently, more precise, personalized strategies that target the root cause of disease without compromising safety or cost-effectiveness are needed. Preclinical *in vivo* studies have confirmed that gene therapy using siRNA can achieve this goal (Sajid et al. 2023). In clinical trials (NCT05231785), Lexeo Therapeutics has conducted gene therapy studies for *APOEε4* allele patients, attempting to convert the brain's APOE protein subtype from APOE4 to APOE2 via intrathecal injection of an adeno-associated virus vector (LX1001) carrying APOE2 cDNA (ClinicalTrials.gov. NCT03634007 2025). Alnylam has developed ALN-APP, an RNAi drug that targets *APP*, which uses proprietary C16-siRNA conjugation technology to enhance CNS delivery, with related patents filed (Brown et al. 2023; ClinicalTrials.gov. NCT05231785 2025). The most significant obstacle to efficient siRNA delivery to the brain remains the BBB. As innovative delivery tools, NPs have been shown in preclinical studies to carry modified siRNA across the BBB for effective gene silencing (Li et al. 2024). Although early patents (e.g. AU 2004263884) have explored using nanocapsules or liposomes to deliver siRNA for AD treatment, this strategy has not yet entered clinical trials (Jin et al. 2025). Its successful translation could provide a novel, precision-targeted treatment option for AD.

#### 4.2.4 Nanoantioxidants

EGCG, a natural antioxidant, has shown potential in inhibiting Aβ aggregation and countering oxidative stress in AD animal models (Guo et al. 2023). A phase III clinical trial explored the effects of EGCG in patients with MCI (NCT00951834) (ClinicalTrials.gov. NCT00951834 2025). Compared to exogenous small-molecule antioxidants, nanoantioxidants offer superior metabolic stability, bioavailability, and BBB penetration. The ‘Natural antioxidants’ section of this article details the efficacy of various nanoantioxidants in preclinical studies. Although clinical trials of nanoantioxidants for AD are not yet widespread, related patents exist (e.g. CN118021741A for ellagic acid crystal NPs as anti-AD drugs), indicating their potential as viable nanomedicines.

#### 4.2.5 Nanoformulations of novel α-secretase modulators

α-Secretase cleaves APP into nontoxic, soluble fragments, representing a potential target for reducing Aβ production. Research indicates that molecules such as retinoic acid can enhance α-secretase activity by activating disintegrin and metalloproteinase domain-containing protein 10 (Peron et al. 2018). An early clinical trial (NCT00880412) evaluated the oral α-secretase stimulant etazolate as an adjunctive therapy (Vellas et al. 2011; Singh et al. 2022), but oral administration is associated with gastrointestinal side effects and low BBB penetration. To address this, researchers have developed APH-1105, a liposome-encapsulated nanomedicine that is administered intranasally and has entered phase II clinical trials (NCT03806478) to assess its safety, tolerability, and efficacy in patients with mild-to-moderate AD (Taléns-Visconti et al. 2023; ClinicalTrials.gov. NCT03806478 2025). Another patent discloses a combination therapeutic strategy employing biodegradable polymer nanospheres coencapsulating keratin and retinol. This synergistic formulation is capable of upregulating α-secretase expression and concomitantly mitigating the deposition of Aβ plaques (US 10828276). These advances highlight the significant advantages of nanomedicines in optimizing administration routes and enhancing brain targeting, positioning them as promising candidate strategies in current clinical-stage AD.

### 4.3 Clinical transformation challenges and future directions

Clinical trials for AD treatment continue to advance, with the pipeline expanding beyond traditional targets to encompass novel mechanisms such as neuroinflammation and gut‒brain axis modulation (Cummings et al. 2025). However, a striking translational gap persists: while numerous nanomedicine platforms show preclinical promise for CNS delivery, very few have entered clinical trials explicitly for AD. This translational gap is not coincidental. It arises directly from a series of interconnected bottlenecks that halt promising candidates at the preclinical stage. To understand these bottlenecks, including scientific, manufacturing, regulatory, and commercial challenges, is essential to bridging the ‘valley of death’ between bench and bedside. Overcoming this requires a paradigm shift in how these complex therapeutics are designed, evaluated, and scaled.

#### 4.3.1 Biological heterogeneity and personalized delivery

AD patients exhibit significant heterogeneity in genetic background, pathophysiology, and clinical presentation, which directly impacts nanomedicine efficacy. Individual differences in disease subtypes, Aβ and tau deposition patterns, neuroinflammation levels, and BBB integrity can lead to considerable variation in how nanomedicines are distributed, targeted, and how effective they ultimately are. While nanotechnology enables personalized medicine through biomarker-customizable formulations, it also introduces significant challenges. These include the need for companion diagnostics, complex manufacturing processes, and sophisticated patient stratification for clinical trials. Many current trial designs do not adequately account for this heterogeneity, potentially diluting overall efficacy results and obscuring the identification of responsive patient subgroups. Advances in artificial intelligence (AI) and machine learning may help address these issues by improving biomarker sensitivity, enabling precise patient stratification, and enhancing disease prediction models, thereby increasing the personalized application value of nanomedicine (Gao et al. 2024; Hassan et al. 2025).

#### 4.3.2 The enduring challenge of the BBB

For a long time, the development of effective drugs for treating CNS diseases has been plagued by a high failure rate in clinical trials. The main obstacle is the restrictive permeability of the BBB to most drugs, which leads to insufficient brain accumulation and suboptimal therapeutic effects. Although surface modifications of NPs can enhance penetration, the long-term, repeated dosing required for chronic conditions such as AD may lead to unpredictable changes in biodistribution, inflammatory responses, or immunogenicity. This is a particular concern for some inorganic nanomaterials, whose nonbiodegradability raises questions about long-term cumulative toxicity (Zhu et al. 2018).

Researchers have innovatively attempted to bypass the BBB by utilizing immune channels adjacent to the brain (i.e. craniomeningeal channels), loading drugs onto NPs to hijack intracranial immune cells, achieving enhanced targeted drug delivery and treating MCAO models. This approach has been applied in clinical trials to treat patients with malignant middle cerebral artery infarction (NCT05849805), demonstrating certain feasibility and safety, highlighting its translational potential (Gao et al. 2026). However, this clinical method requires drilling through the skull, which, although feasible, remains invasive. Therefore, future research should focus on optimizing the immediate penetration efficiency and systematically evaluating long-term neurotoxicity, brain retention kinetics, and the stability of BBB traversal under chronic dosing conditions. Integrating AI-driven predictive models to optimize NP design is a promising strategy to increase brain targeting efficiency and safety.

#### 4.3.3 Ethical considerations in clinical trials

Conducting trials in an AD population compounds standard ethical challenges. The core issue is managing uncertainty in the risk‒benefit ratio (Tinkle et al. 2014). Patients with cognitive impairment are a vulnerable group, and the lack of long-term safety data for novel nanomaterials complicates obtaining truly informed consent. Ethically sound practice demands transparent disclosure about the nanoformulation and its potential unknown risks, even if this may hinder recruitment. Furthermore, early-phase trials in advanced patients may offer minimal direct benefit, requiring researchers to meticulously manage expectations and justify the risk burden (Satalkar et al. 2016). Ethical oversight must also consider potential risks to trial personnel and the environment from exposure during manufacturing or administration (Wolf 2012).

#### 4.3.4 Slow progress in clinical trials: the preclinical attrition

Clinical trials for AD therapeutics continue to expand, with the pipeline evolving from traditional amyloid-centric targets to encompass neuroinflammation, synaptic protection, and the gut‒brain axis (Cummings et al. 2025). However, a pronounced translational disconnect persists: although hundreds of nanomedicine platforms have demonstrated the ability to cross the BBB and modulate AD pathology in animal models, only a select few have advanced to clinical investigations for central nervous system indications. For instance, Patisiran—a LNPs-formulated siRNA targeting transthyretin—was approved for hereditary transthyretin-mediated amyloidosis (hATTR) polyneuropathy following a Phase III trial (NCT01960348). This trial demonstrated significant functional improvement and good safety profiles (Quan et al. 2023). Conversely, the gold nanocatalyst CNM-Au8 failed to slow ALS progression after 24 weeks in the RESCUE-ALS trial (NCT04098406), though long-term follow-up suggested a mortality benefit, warranting further study (Vucic et al. 2023). However, clinical nanomedicine trials specifically for AD remain exceedingly scarce. Beyond the fundamental challenges of BBB penetration and ethical considerations, the frequent failure to translate promising *in vitro* and early animal data is compounded by a cascade of interconnected issues:

First, advancing nanomedicines into clinical trials without a comprehensive understanding of their composition, physicochemical properties, pharmacokinetics, and therapeutic activity increases the risk of costly late-stage failure. Second, variations in dosage, materials, and manufacturing steps during preclinical development can profoundly affect performance. Early identification of these parameters are critical for selecting scalable production methods and establishing process controls to ensure product reproducibility. Third, *in vitro* systems and animal models often fail to reliably predict nanomedicine behavior in humans. Absorption, distribution, metabolism, and excretion may vary with administration route or disease stage, revealing bio-nano interactions not captured in simplified systems (Younis et al. 2022). Finally, under systemic administration, NPs rapidly adsorb plasma proteins (e.g. albumin), forming a protein corona that alters surface charge, aggregation, and immune recognition. This can trigger immune toxicity while reducing targeting specificity, thereby complicating the predictability of *in vivo* drug delivery.

#### 4.3.5 Translation bottlenecks

The translational rate for nanomedicines from bench to bedside is dismally low, typically less than 10% (Adams 2012). This high preclinical attrition rate is a direct consequence of profound and unresolved translational bottlenecks.

The clinical translation of nanomedicines for AD faces multidimensional bottlenecks (Hossain and Hussain 2025; Zahariev et al. 2025). First, at the technical level, major hurdles include the complex *in vivo* behavior of NPs and the difficulty in maintaining consistent performance during scale-up, a challenge compounded by the inherent batch-to-batch variability that makes standardized testing and quality control exceptionally difficult (Stiufiuc and Stiufiuc 2024). Second, at the regulatory and manufacturing level, a lack of standardized protocols for assessing long-term toxicity, coupled with the challenge of developing large-scale, low-cost, and highly reproducible manufacturing processes that meet regulatory standards, remains significant (Joyce et al. 2024). Third, at the financial level, the substantial investment required from preclinical development through clinical trials often exceeds the resources of academic institutions. Industry partners may be cautious due to the high risk and long return on investment. Finally, at the public perception level, public awareness and concern about the potential risks of nanotechnology can influence policy support and clinical acceptance (Manikkath et al. 2017).

Ultimately, overcoming preclinical attrition depends on addressing the entire spectrum of bottlenecks as a cohesive system rather than as isolated challenges. Establishing standardized preclinical protocols for safety, biodistribution, and therapeutic effect assessment is urgently needed to harmonize global research and build a stronger foundation for human trials (Adams 2012; Younis et al. 2022).

#### 4.3.6 The Regulatory imperative: from technical challenges to development requirements

The scientific and manufacturing bottlenecks described above converge at a critical juncture: the regulatory evaluation. Agencies such as the FDA and European Medicines Agency (EMA) require unequivocal proof of safety, quality, and efficacy precisely because of the complex *in vivo* behavior and manufacturing challenges inherent to nanomedicines. Therefore, navigating the regulatory perspective becomes paramount, as it translates these technical hurdles into specific, mandatory development requirements.

Compared to conventional drugs, which typically rely on a single active agent, most nanomedicines exhibit inherently complex physicochemical and biological properties. However, current drug approval frameworks were primarily developed for traditional, simple drug molecules. Given the current lack of clear and specific guidelines for evaluating complex, multifunctional, or theranostic nanosystems, there is an urgent need to establish standardized protocols and regulatory frameworks specific to nanomaterials to enable their efficient evaluation and approval (Mühlebach 2018; Kardani 2024). Regulatory agencies require exhaustive characterization of a nanomedicine's physicochemical properties and pharmacokinetics. Specifically, for a nanomedicine product, a chemical, manufacturing, and control (CMC) package is needed and typically includes particle size, morphology, drug loading, *in vitro* release, surface characteristics/coating characteristics, porosity, stability, sterility, endotoxin level, and pyrogenicity (Clogston et al. 2024). What's more, given that nanomedicines are nonbiological complex drugs, batch-to-batch variability can directly affect their efficacy or introduce nanotoxicity risks. Consequently, FDA and EMA impose stringent requirements on batch reproducibility for generic nanomedicines, necessitating comprehensive quality comparison data and the use of multiple complementary analytical methods to ensure consistency. The application of quality by design (QbD) principles throughout the development process is recommended to increase batch reproducibility (Csóka et al. 2021). In addition, a major safety concern is the chronic toxicity arising from off-target NP accumulation in peripheral organs (e.g. liver and spleen), which not only reduces brain bioavailability but also poses long-term health risks. Consequently, regulatory agencies mandate comprehensive data beyond routine requirements, including detailed evidence on *in vivo* biodistribution, metabolic pathways, immunogenicity, and long-term organ-specific toxicity (Wang et al. 2025).

Overcoming these bottlenecks will require deep interdisciplinary collaboration among academia, industry, and regulators; coordinated public and private investment to share risks; and ultimately, the establishment of an internationally harmonized regulatory and long-term safety monitoring framework adapted for chronic conditions such as AD.

## 5 Conclusion

AD persists as a critical global health threat to aging populations. Accelerated demographic aging, particularly evident in the population structure of China, continues to elevate the prevalence of dementia. This trend generates substantial socioeconomic burdens through healthcare expenditures while imposing profound physical and psychological challenges on affected families and caregivers. Consequently, developing effective AD therapeutics represents an urgent medical priority.

However, current therapeutic strategies demonstrate significant limitations in addressing the complex pathology of AD. Existing approaches inadequately target the multifactorial nature of the disease, which involves interconnected mechanisms, including cerebral Aβ aggregation and tau protein hyperphosphorylation. Immunotherapies remain insufficiently nuanced for such intricate neurological disorders. Furthermore, approved AD medications (e.g. donepezil and galantamine) provide merely symptomatic relief while exhibiting notable drawbacks: adverse effects, limited bioavailability, and poor BBB penetration.

In this review, we highlight that the expanding application of nanotechnology has positioned nanomedicine as a promising diagnostic and therapeutic strategy for neurodegenerative disorders. Specifically, NP delivery systems enhance BBB penetration by virtue of their high biocompatibility, electrostatic interactions, and hydrophobic properties. After administration (e.g. intranasal delivery), these systems achieve targeted drug delivery and controlled release via multiple stimulus-responsive mechanisms, efficiently transporting therapeutic agents (e.g. antioxidants, neurotrophic factors) to neural tissues.

Notably, AD pathogenesis involves multiple genetic factors, including key genes such as *APP*, *PSEN1*, *PSEN2*, and the high-risk variant *APOEε4* allele. *BACE1* is further implicated in Aβ accumulation. Beyond the mainstream Aβ cascade and tau hyperphosphorylation, studies indicate causal links between AD and neuroinflammation, oxidative stress, cholinergic dysfunction, gut microbiota dysbiosis, and estrogen deficiency. Thus, researchers are developing intelligent nanomedicine systems for multidimensional AD intervention, including targeted gene therapy, the inhibition of Aβ/tau aggregation, and the mitigation of oxidative damage.

Nevertheless, the clinical translation of nanomedicines faces challenges. Preclinical studies indicate favorable biosafety and minimal cytotoxicity, yet most evidence is derived from rodent models. Further investigations are needed to assess potential excitotoxicity, DNA damage, and functional toxicity. Additionally, while the size/surface properties of NPs enhance BBB penetration, they present limitations such as suboptimal drug-loading capacity, saturation risks, and aggregation-induced blockage of active sites. Therefore, future research must develop structurally optimized and functionally smarter nanodrugs to achieve clinical applications, including disease-modifying therapies or early diagnosis.

Overall, the treatment of AD has progressed from ‘symptomatic relief’ to ‘etiological treatment’. Considering the heterogeneity of AD patients, appropriate methods should be selected at each stage, such as nanomaterial design, loaded active ingredients, carrier selection, and stimulus-responsive drug release, to target the corresponding pathogenic mechanisms for specific treatment, achieve a comprehensive nanomedicine-based diagnosis and treatment system and provide new strategies for early intervention and individualized treatment of AD. Additionally, interdisciplinary integration should be promoted, especially the integration of AI and machine learning into nanomedicine research. AI-guided nanocarrier design enables high-throughput simulation and optimization of carrier physicochemical properties (e.g. size, shape, and surface ligands). This allows for precise prediction of BBB penetration efficiency, targeting binding ability, and in vivo distribution of nanocarriers. The predicted results further guide the synthesis of more intelligent and efficient next-generation nanodrugs. Furthermore, a set of forward-looking and systematic safety assessment frameworks for nanodrugs must be established and improved. The academic community, industry, and regulatory agencies should collaborate to develop standardized characterization and testing guidelines for multifunctional nanodrugs (especially integrated diagnostic and therapeutic formulations). Through the dual drive of technological innovation and prudent evaluation, nanomedicine can truly overcome the bottleneck of transformation and achieve its ultimate promise for diagnosing and treating AD.

## Data Availability

Data sharing is not applicable to this article as no new data were created or analyzed in this study.
